# The Distribution of Glucosinolates in Different Phenotypes of *Lepidium peruvianum* and Their Role as Acetyl- and Butyrylcholinesterase Inhibitors—In Silico and In Vitro Studies

**DOI:** 10.3390/ijms23094858

**Published:** 2022-04-27

**Authors:** Dominik Tarabasz, Paweł Szczeblewski, Tomasz Laskowski, Wojciech Płaziński, Ewa Baranowska-Wójcik, Dominik Szwajgier, Wirginia Kukula-Koch, Henry O. Meissner

**Affiliations:** 1 Department of Pharmacognosy with Medicinal Plants Garden, Medical University of Lublin, 1 Chodzki Str., 20-093 Lublin, Poland; dominikanes@o2.pl; 2 Department of Pharmaceutical Technology and Biochemistry and BioTechMed Centre, Faculty of Chemistry, Gdańsk University of Technology, Gabriela Narutowicza Str. 11/12, 80-233 Gdańsk, Poland; pawel.szczeblewski@pg.edu.pl (P.S.); tomasz.laskowski@pg.gda.pl (T.L.); 3 Jerzy Haber Institute of Catalysis and Surface Chemistry, Polish Academy of Sciences, Niezapominajek 8, 30-239 Krakow, Poland; wojtek_plazinski@o2.pl; 4 Department of Biopharmacy, Faculty of Pharmacy, Medical University of Lublin, Chodźki 4A, 20-093 Lublin, Poland; 5 Department of Biotechnology, Microbiology and Human Nutrition, University of Life Sciences in Lublin, Skromna 8 Street, 20-704 Lublin, Poland; ewa.baranowska@up.lublin.pl (E.B.-W.); dominik.szwajgier@up.lublin.pl (D.S.); 6 Therapeutic Research, TTD International Pty Ltd., 39 Leopard Ave., Elanora, Gold Coast 4221, Australia; dr.meissner@ttdintnl.com.au

**Keywords:** maca tubers, PCA, modelling, HPLC-MS, AChE and BuChE inhibition

## Abstract

The aim of the study was to present the fingerprint of different *Lepidium peruvianum* tuber extracts showing glucosinolates-containing substances possibly playing an important role in preventinting dementia and other memory disorders. Different phenotypes of *Lepidium peruvianum* (Brassicaceae) tubers were analysed for their glucosinolate profile using a liquid chromatograph coupled with mass spectrometer (HPLC-ESI-QTOF-MS/MS platform). Qualitative analysis in 50% ethanolic extracts confirmed the presence of ten compounds: aliphatic, indolyl, and aromatic glucosinolates, with glucotropaeolin being the leading one, detected at levels between 0–1.57% depending on phenotype, size, processing, and collection site. The PCA analysis showed important variations in glucosinolate content between the samples and different ratios of the detected compounds. Applied in vitro activity tests confirmed inhibitory properties of extracts and single glucosinolates against acetylcholinesterase (AChE) (15.3–28.9% for the extracts and 55.95–57.60% for individual compounds) and butyrylcholinesterase (BuChE) (71.3–77.2% for the extracts and 36.2–39.9% for individual compounds). The molecular basis for the activity of glucosinolates was explained through molecular docking studies showing that the tested metabolites interacted with tryptophan and histidine residues of the enzymes, most likely blocking their active catalytic side. Based on the obtained results and described mechanism of action, it could be concluded that glucosinolates exhibit inhibitory properties against two cholinesterases present in the synaptic cleft, which indicates that selected phenotypes of *L. peruvianum* tubers cultivated under well-defined environmental and ecological conditions may present a valuable plant material to be considered for the development of therapeutic products with memory-stimulating properties.

## 1. Introduction

According to available statistics, average life expectancy and longevity increased from about 45 years in 1900 to over 75 years today. This can be attributed to advances in medical, biochemical, nutritional, and environmental sciences which, in turn, constantly contribute to better medical treatment and socially implemented progress in living conditions and extended life expectancy. 

The extension of human life span over the last century has translated into an increase in the incidence of diseases that disrupt the work of the Central Nervous System (CNS), especially in highly developed countries. Neurodegenerative diseases are considered a global epidemic due to their prevalence and constantly increasing incidence rates. According to data reported by the World Health Organization (WHO), in 2050, the number of people with dementia is expected to exceed 115 million worldwide [1]. Therefore, the search for new active compounds with neuroprotective activity is of the highest importance. So far, several natural products have been introduced to the treatment of neurodegenerative disorders. Galantamine, an isoquinoline alkaloid from *Amaryllidaceae*, is certainly a well-known example of a drug introduced to the therapeutic strategies in the treatment of the symptomatic Alzheimer’s disease (AD) as an acetylcholinesterase (AChE) inhibitor [2]. This group of medicines regulates the level of acetylcholine (ACh) in the synaptic cleft. It was proved that during the aging of the organism, the amount of ACh decreases, and additionally, an increase in the activity of esterases (acetylcholine- and butyrylcholinesterase (BuChE) that decompose ACh is observed that decreases the effectiveness of stimuli transmission. For that reason, the esterase inhibitors are first-line drugs that improve the transmission of the stimulus between neuronal cells [3]. The drugs available on the market, however, have a number of side effects or an insufficient duration of action, as discussed in comprehensive reviews, e.g., by Haake et al. 2020 [4]. Therefore, scientists continuously search for other medicines that could be more efficient in regulating the process described above.

During the last 25 years, one of the plants intensively researched for its influence on a wide spectrum of health and medical conditions contributing to improved gender-specific healthy living status and extended longevity is a tuber (or hypocotyl) of a native plant from the Brassicacae botanical family—Peruvian maca. This plant represents one of some 175 identified *Lepidium* species—*Lepidium peruvianum* synonym *L. meyenii* is known under the common name maca. In this work, Peruvian maca will be referred to as *L. peruvianum* [5]. When harvested in the high Andes, the maca crops are composed of some 13 differently coloured hypocotyls termed in this work as “phenotypes”, with yellow, red, black, and purple appearing to be dominant [6].

Maca has a long history of cultivation and consumption since the time of the Incas established in its acknowledged native habitat—the Junín plateau in the highlands of the Peruvian Andes [7]. Now it is commercially grown as an important cash crop and therapeutic commodity for domestic use and for export [8]. Blends or individual maca phenotypes, distinguished by hypocotyls’ colour, have induced different gender- and age-specific physiological responses, alleviating the severity of several known health conditions [9,10,11]. When taken orally as a dietary supplement, Peruvian maca is considered a non-hormonal, adaptogenic herb exhibiting hormone-regulating properties for a wide range of age groups of men and women [6,12,13], helping to restore metabolic harmony and induce hormonal balance specific to gender and appropriate age-stage.

Amongst the well-established multi-pharmacological functions of Peruvian maca, the confirmed antidepressant effect of this plant was found interesting to the authors as it implies the possibility for maca metabolites to display the ability to penetrate the blood–brain barrier [14].

During the last 30 years, Peruvian maca hypocotyls were the subject of extensive chemical and nutritional studies [6,7,15,16], with glucosinolates (GLSN) identified as the main active natural constituents and glucotropaeolin (GLT) and methoxyglucotropaeolin (MGT) as the leading components [16,17,18]. Additionally, unsaturated fatty acids and benzylated alkamides (macamides and macaenes) have been detected as unique to maca, helping in chemical identification of this plant [18,19]. 

Glucosinolates are well-defined biochemical plant components belonging to a group of metabolites characterized by the presence of nitrogen and sulphur atoms in their structure. They are spread across the representatives of the following botanical families: *Brassicaceae*, *Tovariaceae*, *Capparidaceae*, *Stegnospermaceae*, *Moringaceae*, *Resedaceae*, and other unrelated plant families, such as *Limnanthaceae*, *Caricaceae*, and *Tropaeolaceae*. When the structure of plant cells is physically disrupted or damaged, glucosinolates are hydrolyzed by the enzyme called myrosinase, which usually occurs during the physical processing of tubers. As a consequence, a sugar molecule is released, whereas the aglycon degrades, forming oxazolidine-2-thiones, nitriles, isothiocyanates, epithionitriles, thiocyanates, and other derivatives of interest from a pharmacological perspective [20,21]. Recent studies confirm the broad medicinal properties of glucosinolates and isothiocyanates that include anticancer and antimicrobial activity [22] and provide protection to the cardiovascular and central nervous systems, which may be important in the prevention of diabetic nephropathy and neuropathy [23].

Only a few up-to-date publications dealt with the anticholinesterase properties of this group of secondary metabolites. Previously, Burcul and colleagues determined the inhibitory properties of 11 compounds from the isothiocyanate group. Of these, 2-methoxyphenyl isothiocyanate showed the best inhibitory properties against AChE with IC_50_ of 0.57 mM, while in the BuChE inhibition test, 3-methoxyphenyl isocyanate showed the best result of 49.2% at 1.14 mM [24]. Additionally, Blazevic et al. [25] investigated volatile isolates from *Bunias erucago*, with glucosinalbin and glucoraphanin as the leading components inhibiting both AChE and BuChE. Two components of extracts from maca tubers, namely N-benzylhexadecanamide and N-acetylbenzylamine, were already reported to exhibit neuroprotective effects, presumably partly due to inhibition of the AChE and BuChE enzymes [26], but the scientific literature still lacks information on the potential of glucosinolates from maca.

This fact encouraged the authors to embark on further study to assess inhibitory properties of glucosinolates present specifically in Peruvian maca (*Lepidium peruvianum* syn. *L. meyenii*) and to explain their effect towards the AChE and BuChE enzymes. Another reason for choosing this plant for the study is the fact that Peruvian maca has been demonstrated to contain about 100 times more glucosinolates than other well-known cruciferous plants, such as cabbage or broccoli [14]. 

These goals are complemented by a desire to compare the content of glucosinolates and to study the fingerprints of the rich collection of five prime Peruvian maca phenotypes labelled as “Yellow”, “Black”, “Red”, “Purple”, and “Gray” that were grown in two locations of the Peruvian Andean Highlands. In this research, the HPLC-ESI-QTOF-MS/MS technique and the chemometric approach were adopted to analyse the obtained results, which were extended for measuring the in vitro inhibitory potential of individual glucosinolate fractions towards AChE and BuChE. Additionally, the explanation of the grounds for the memory-enhancing activity of glucosinolates using an in silico approach linked to modeling studies was undertaken and was specifically adopted in an attempt to explain the dynamics behind utilizing the inhibitory potential of these metabolites towards both the AChE and BuChE enzymes.

## 2. Results and Discussion

### 2.1. Qualitative Analysis of Extracts from Lepidium peruvianum by Liquid Chromatography Coupled with Mass Spectrometry (HPLC-ESI-QTOF-MS/MS)

**Figure 1:**
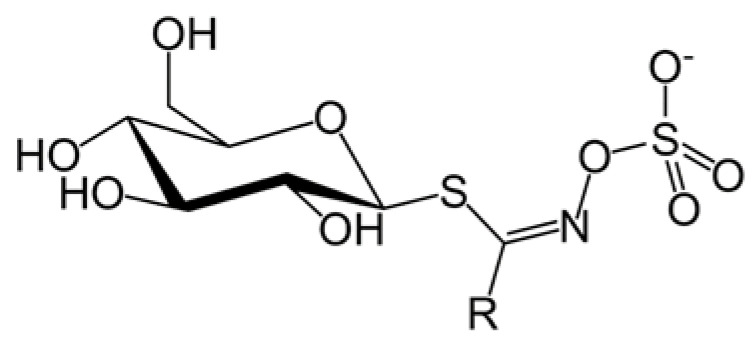
General structure of glucosinolates, the group R is variable.

Glucosinolates (Figure 1) are composed of a glucose structure which is combined with an O-sulfated thiohydroximate moiety and are variable in length—side chain is variable in derivatives of amino acids. The aliphatic chain is biosynthesized most often from methionine, aromatic moiety from phenylalanine or tyrosine, and the indole components from tryptophan [27,28]. Isothiocyanates are formed during the hydrolysis of glucosinolates. Using the example of glucotropaeolin (GTP), we will follow the stages of hydrolysis and the end products. During the hydrolysis of GTP with the participation of myrosinase, the glucose molecule and the hydroxysulfate ion are detached from the structure. As a result, a biologically active compound called benzyl isothiocyanate is formed, which is the major intermediate to benzylamine formation [29]. An understanding of the mechanisms of glucosinolates transformation is essential to determine their presence in plant extracts. 

The applied chromatographic conditions allowed for the determination of twenty metabolites in the extracts of *L. peruvianum* that were visible in the negative ionization mode (also see Figures S1 and S2 in the Supplementary File), as presented in Table 1. The identification was based on high-resolution molecular weight measurements, analysis of their MS/MS spectra, retention times, open MS databases, and scientific literature and is in line with the previously published data of other researchers [14,26,30]. The total ion chromatogram (see Figure S3 in Supplementary File) of the selected extract, the extracted ion chromatograms of all glucosinolates whose presence was confirmed in the negative mode [26], and the MS/MS spectra of the tentatively identified structures are presented in Table S1 in the Supplementary File. 

The obtained data clearly indicate that glucosinolates are the major constituents visible in the negative ionization mode in the majority of the tested samples. Among them, glucotropaeolin (GTP, **G2**)—an aromatic glucosinolate—eluted in the ninth minute was the major peak. Additionally, other representatives from different groups of sulphur-containing compounds were identified in the fingerprints of maca 50% EtOH (*v*/*v*) extracts: 1-isothiocyanato-9-methanesulfinylnonane (**G6**); aromatic glucosinolates, such as glucosinalbin (**G1**); and glucolimnanthin (**G3**). Indolyl glucosinolates: methoxyglucobrassicin (**G4**), 4-methoxyindolyl-3-hexylhydroxyglucosinolate (**G5**), and indolyl-3-hexyl-4-methyl-cyclohexaneglucosinolate (**G9**). Aliphatic glucosinolates: glucoalyssin (**G8**) and pent-4-enylglucosinolate (**G7**). Moreover, the HPLC-ESI-QTOF-MS/MS analysis revealed the presence of several organic acids, such as gluconic, malic, citric, succinic, and phenolic acids; benzoic acid and its derivatives; tannins like fucodiphlorethol; and fatty acids represented by trihydroxy-(3)-octadecadienoic acid and pinellic acid.

### 2.2. Chemometric Assessment

#### 2.2.1. Data Preparation

Glucosinolates were found to constitute the leading role in the analyzed *L. peruvianum* extracts. Based on the qualitative determination of the extracts’ constituents presented in the Table 1, the further aim of the study was to compare the content of glucosinolates and to analyse their relative concentration in the extracts. 

For that reason, the chemometric analyses of the presented dataset of *L. peruviaum* extracts were prepared in order to compare absolute (**ABS**) and relative (**REL**) glucosinolate compositions of the studied samples. For this purpose, the peak areas of nine compounds (**G1–G9**, see Table 1) were collected in triplicate from the recorded chromatograms of every extract injected into the HPLC-MS instrument. For further information on data preparation, please consult Materials and Methods (Section 3.4).

#### 2.2.2. The “Absolute” Approach (**ABS**)

Principal component analysis (**PCA**) performed for the **ABS** dataset (Table S2 in the Supplementary File) resulted in the selection of the first three principal components (**PCs**), explaining 79.8% of the total variance (Table S3 in the Supplementary File). After the VARIMAX rotation, the following variables were associated with the resulting dimensions: (1) first dimension (**Dim1**)—**G1**, **G2**, **G3**, **G8**; (2) second dimension (**Dim2**)—**G4**, **G9**; (3) third dimension (**Dim3**)—**G6**, **G7**. The remaining **G5** variable constituted the fourth dimension of the studied dataset, yet it was not included in the further analyses (Table S4 in the Supplementary File).

**Figure 2:**
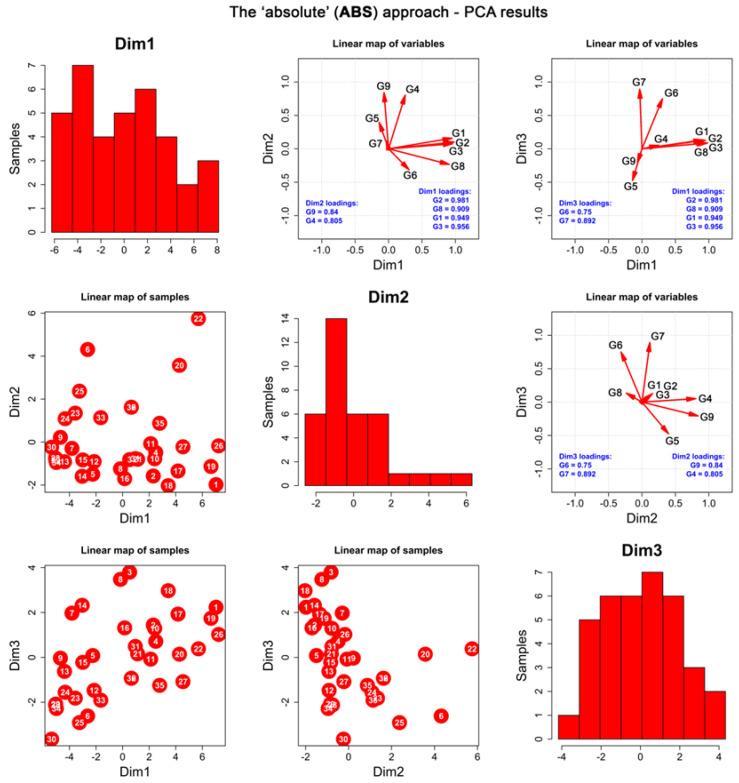
**PCA** performed for the **ABS** dataset. Diagonal: histograms of the first three resulting **PCs** subjected to VARIMAX rotation (henceforth known as dimensions, **Dim**). Lower triangle: linear maps of samples in the space of the first three dimensions. Upper triangle: linear maps of original variables (compounds) in the space of the first three dimensions. The numbers correspond to the numbering presented in the Section 3.1.

**PCA** and the correlation analysis (Figure 2 and Figure S4) have shown that the absolute extraction ratios of aromatic glucosinolates (**G1**, **G2**, **G3**) and aliphatic glucoalyssin (**G8**), constituting **Dim1**, were strongly correlated. This means that the content of **G1**, **G2,** and **G3** is related to the content of **G8**. The higher content of the aromatic glucosinolates in the extracts of maca, the higher the amount of **G8**.

**Figure 3:**
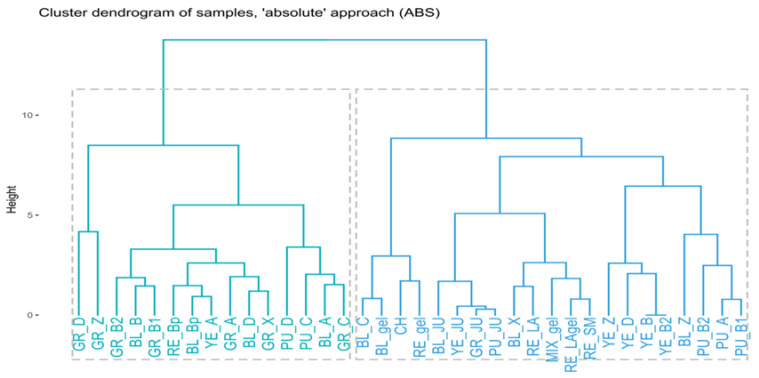
Cluster dendrogram prepared on the basis of the **ABS** dataset.

It is worth noting that the first dimension (**Dim1**), represented by a bimodal distribution, was responsible for the division of the dataset into two subsets of samples, which was also clearly demonstrated by the hierarchical clustering (**HC**, Figure 3). The obtained results classify the tested samples into two different groups—one of which collects juice samples obtained from fresh tubers, technologically altered gelatinized samples, maca tubers collected in the Ancash area, and yellow and purple tubers that seem to have different profiles from the remaining phenotypes—whereas the other collects large tubers of differently coloured phenotypes, mostly collected from Junin location.

Linear maps of samples (Figure 2), combined by the cluster dendrogram resulting from **HC** (Figure 3), have revealed that juices contained very similar amounts of glucosinolates, with BL_JU (No 7) slightly differentiating from the rest (Figure 3). These observations are in line with previous findings on the formation of glucosinolates in the plant tissues that underline that the process of oxidation is necessary to trigger the conformational changes in their structures [27]. Juices that were squeezed out from fresh tubers and immediately analysed did not contain high quantity of glucosinolates yet.

Moreover, the following samples collected in mid-October 2017: MIX_gel-AN, BL_gel-AN, BL_Bp-AN, RE_Bp-J, RE_gel-AN, RE_SM, RE_LA, and RE_LAgel contained similar amounts of glucosinolates (Figure 3). Interestingly, gelatinized samples in general contained low amounts of aromatic glucosinolates and **G8** (**Dim1**) in comparison with non-gelatinized tubers, except for BL_gel-AN (located at the middle of the **Dim1** axis). However, they all exhibited similar, medium ratios of **G4**, lower than unprocessed samples. 

As a raw material containing a large quantity of starch, in order to improve their nutritional properties, maca tubers are subjected to a pretreatment process—gelatinization. The results presented above show that this process, which takes place at an elevated temperature, induces thermal decomposition of glucosinolates because their content in gelatinized samples is lower in relation to the untreated samples. 

In the study, the composition and content of glucosinolates was found to be colour- and size- dependent. As previously mentioned, the above presented dendrogram (Figure 3) classifies the analysed extracts into two major groups. Interestingly, a majority of the extracts from black, grey, and red phenotypes tend to show a similar composition, whereas the extracts from yellow and purple phenotypes are more distant from other samples in terms of glucosinolate content. Specifically, small, grey tubers (GR_D-J, GR_Z-J) contained higher amounts of indolyl glucosinolates, i.e., **G4** and **G9** (**Dim2**) than the rest of the tubers. Large red, grey, and black tubers (A, B, X) were poor in **G4** and **G9**, in contrast to the small ones (for which the trend was not applicable to purple and yellow species). Red tubers in general contained low amounts of indolyl glucosinolates (**Dim2**), while all the red species (excluding RE_Bp-J) contained smaller quantities of glucosinolates and **G8**, constituting the **Dim1**. Red and purple tubers produced lower amounts of **G6** and aliphatic glucosinolate **G7** (**Dim3**). On the contrary, black tubers excelled in ratios of **G6** and **G7** produced, with the exclusion of juiced samples (BL_JU) and extra small phenotypes (BL_Z-J). Generally, large tubers seem to differ in composition in comparison to small tubers, which can be seen in the above dendrogram. The glucosinolates’ profiles of yellow and purple phenotypes are alike. These results are in accordance with the study of Clement et al. [41], who calculated total glucosinolate content in their work on four phenotypes of maca. In their study, the grey phenotype was found to contain the highest amount of total glucosinolates (54.78 μmol/g d.w.), followed by the red (44.10 μmol/g d.w.), yellow (37.23 μmol/g d.w.), and purple (33.22 μmol/g d.w.) phenotypes. For that reason, the maca phenotypes tested herein are divided into two groups—one containing black, grey, and red tubers and another with yellow and purple tubers—seems to be in accordance with former observations on the total glucosinolates content. 

Finally, the chicha morada (CH) species was also poor in **G1**, **G2**, **G3,** and **G8** (**Dim1**), probably due to the fact that they were not dried sufficiently at the place of collection.

#### 2.2.3. The “Relative” Approach (**REL**)

**Figure 4:**
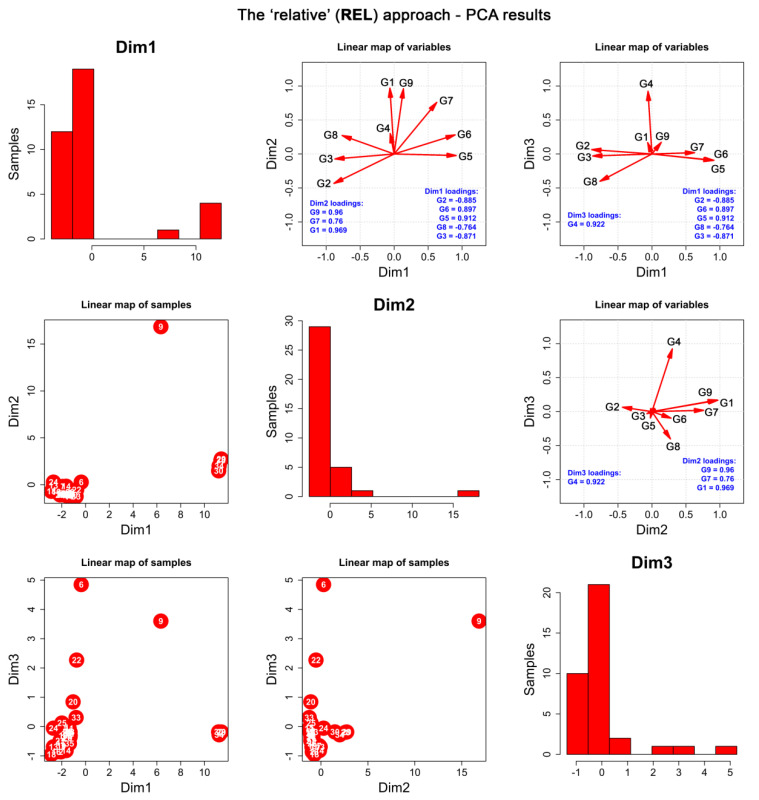
**PCA** performed for the **REL** dataset. Diagonal: histograms of the first three resulting **PCs** subjected to VARIMAX rotation (henceforth known as dimensions, **Dim1**). Lower triangle: linear maps of samples in the space of the first three dimensions. Upper triangle: linear maps of original variables (compounds) in the space of the first three dimensions.

According to the authors’ knowledge, the relative approach to the content of glucosinolates in maca tubers was applied for the first time. This method of data analysis allows for a study on the relations between these metabolites in the maca extracts. Principal component analysis (**PCA**) performed for the **REL** dataset (Table S5 in the Supplementary File) also resulted in selection of three principal components (**PCs**) which explain 90.5% of the total variance (Table S6 in the Supplementary File). Correlation analysis, as well as the VARIMAX rotation, resulted in a linear map of variables in the space of three varivectors, further referred to as “dimensions” (Figure 4 and Figure S5 in the Supplementary File). This revealed that the relative amounts of indolyl glucosinolates **G5** and **G6** were strongly yet reversely correlated with aromatic glucosinolates **G2** and **G3** and indolyl glucosinolate **G8**, while all five compounds constituted the first dimension of the **REL** dataset, **Dim1**. These observations show that the synthesis of the individual groups of glucosinolates shall engage different biosynthetic pathways which are dependent on one another. 

Additionally, aliphatic glucosinolate **G7** was reversely correlated with **G2, G3,** and **G8**, yet **PCA** revealed its stronger connections with indolyl glucosinolate **G9** and aromatic glucosinolate **G1**. Therefore, **G1**, **G7**, and **G9** defined the second dimension of the studied dataset, **Dim2**. The relative ratios of indolyl glucosinolate **G4** solely constituted the third dimension of the **REL** matrix, **Dim3,** which means that the biosynthesis of this compound has no relations to other glucosinolates analysed in this work. This dataset is well explained by a three-dimensional set of factors since no original variables escaped into higher dimensions (Tables S6 and S7 in the Supplementary).

**Figure 5:**
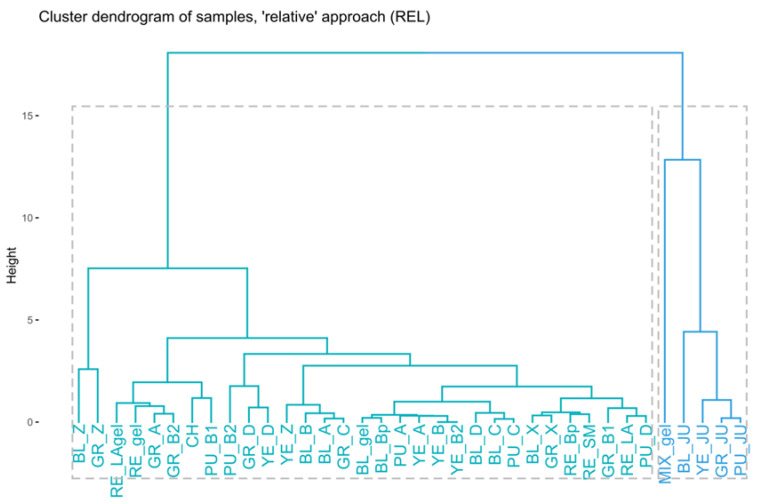
Cluster dendrogram prepared on the basis of the **REL** dataset.

Further **PCA** and **HC** studies (Figure 4 and Figure 5) have shown that the size of the tubers was correlated with the relative composition of the extracts, which additionally confirms the outcomes of the described above **ABS** approach. 

The extracts that are located close to one another are characterized by similar relative proportions of glucosinolates. 

Juices were observed to form a very separated subgroup within the **REL** dataset, with BL_JU being a “black sheep” of the juicy family, as it contains different proportions of the analysed glucosinolates as other samples. This differentiation was observed on the cluster dendrogram and the linear map of samples and mainly resulted from the first dimension (**G2**, **G3**, **G5**, **G6,** and **G8**). Gelatinization did not impact the relative composition of the glucosinolates within the black phenotype family, which explains that the eventual loss in the content of glucosinolates is the same for all compounds. However, the gelatinized MIX_gel-AN sample was a clear outlier in the **REL** dataset, substantially differentiating its relative composition from the rest of the extracts mainly due to its high **Dim1** score (yet lower than juiced) and highest-by-far **Dim2** score (**G1**, **G7,** and **G9**, Figure 4). A high **Dim1** score indicates a marked relative concentration of **G5** and **G6**, but low **G2**, **G3**, and **G8**, whereas the high **Dim2** score of the sample is induced by increased relative contents of **G1**, **G7**, and **G9** in comparison to other glucosinolates. Therefore, MIX_gel-AN was also the last sample to join the cluster dendrogram with a Euclidean distance above ~13 (Figure 5), which indicates that this sample exhibited extremely different relative composition in comparison to the others.

The **REL** dataset analysis shows that there are important correlations between certain groups of glucosinolates that may come from the similar, yet competing pathways of their biosynthesis in the plant.

### 2.3. Quantitative Determination of Glucotropaeolin Content in Lepidium peruvianum Extracts Using HPLC-ESI-QTOF-MS/MS

A comparative quantitative analysis of the glucotropaeolin (**G2**) content—the leading glucosinolate of the tested samples—was performed (Table 2) using the HPLC-MS methodology to visualize the existing differences between the analysed extracts. The analysis performed based on triplicate injections determined the content of this major glucosinolate in the 50% (*v*/*v*) alcoholic extract as ranging from 0 to 1.572%. Fresh juice samples did not contain **G2**, whereas its presence was confirmed in all remaining dried tuber samples.

Next, the relationship between the content of **G2** and the color of tubers was investigated. For all five tested varieties of maca—black, purple, grey, yellow, and red—marked differences between the injections were noted. When analyzing the size B of all varieties, grey, red and black maca tubers were found to contain the highest quantity of this compound. The extract from black maca (BL_B-J) contained 0.965% of **G2**; from red maca (RE_B-J), 0.923%; whereas the extract from grey maca (GR_B-J) contained 1.010% (see Table 2). The smallest content of **G2** was calculated for the purple variety: 0.239%. Interestingly, the level of this glucosinolate in the Chica morada (CH) variety was equal to 0.089%.

A drop in **G2** concentration was noted for the gelatinized samples. Black gelatinized tubers (BL_gel-AN) contained 0.624% **G2** (a drop from 0.965%), small gelatinized red tubers contained (RE _gel-AN) 0.103% **G2** (without processing: 0.242%), and large red gelatinized maca tubers (RE_LAgel) 0.061% **G2** (without processing: 0.344%).

Previous studies discuss the content of glucosinolates in maca samples of different origins. It has been observed that batches from different producers and differently coloured phenotypes significantly differed in quantity of glucosinolates. According to Clement et al. [41], the content of glucotropaeolin was the highest in lead-coloured (grey) hypocotyls compared to red, yellow, and purple phenotypes, which is in accordance with the herein presented studies. Another study by the authors [42] confirms an important role of cultivation and cultivation conditions in the biosynthesis of secondary metabolites. The extracts from the same phenotypes of maca (lead-colored, red, yellow, and purple) were examined, but they differed with collection site. The content of **G2** in the lead-coloured phenotype was calculated as 44.23–45.35 µmol/g of dry matter from the Patala area and only 2.37–2.89 µmol/g of dry matter from Alpacayan area. Additionally, our studies confirmed marked differences between the samples collected in the Junín and Ancash areas, with a higher content of these secondary metabolites from the former group.

### 2.4. Inhibitory Activity of Individual Glucosinolates and the Total Extract towards AChE and BuChE

The obtained results confirmed the inhibitory activity of the tested total extracts and the standards. 

Concerning the results of anti-enzymatic activity exhibited by the reference compounds, **G4** was found to be the strongest component. Glucobrassicin derivative showed the best inhibitory properties (enzyme inhibition value: 57.6%) towards AChE among the tested compounds in relation to BuChE, for which the calculated inhibition percentage of **G4** was equal to 39.9% (see, Table 3). Then, in descending order, for AChE **G1** > **G3** > **G2,** with 38.9, 37.05, and 36.2 for BuChE and 56.9, 56.5, and 55.95 for AChE, respectively. It should be underlined that **G4** was the leading enzyme inhibitor, whereas the following compounds were characterized by a similar inhibitory strength.

The results obtained in the tests on reference compounds were set together with the outcomes of the anti-enzymatic assays performed for some selected total extracts. All samples were tested at the concentration of 10 mg/mL, so they can be directly compared with one another. It is important to note that, based on the results of reference compounds, the share of glucosinolates in the total enzyme inhibitory activity can be marked, especially for AChE enzymes. The AChE inhibitory activity of the total extracts ranges from approximately 15–25%, whereas the activities of single glucosinolates are within the range of 55–57%. Based on these observations, they can noticeably influence the total activity of the extract. In the case of BuChE, where the activity of the reference compounds is between 37–40%, the total inhibition rate of the extracts is higher (71–77%), so it must be influenced also by other components present in the extracts.

However, the quantitative data obtained in this study seem to correspond with the results of the enzymatic assay presented herein. Both AChE and BuChE inhibition decrease together with a decrease in size of tubers and with a decrease in the content of glucosinolates, as can be noted in the case of black maca (see Table 3).

Additionally, color-dependent variations were observed between the samples. Black, grey, and red tubers (size B) show a similar anti-enzymatic potential, whereas the yellow variety is both a weaker enzyme inhibitor and a weaker source of glucosinolates.

Rubio et al. reported that aqueous and hydroalcoholic extracts of *Lepidium meyenii* reduced activity of AChE in the brain by 45% after memory impairment caused by scopolamine [43]. Three different maca varieties (black, yellow, and red) were tested to evaluate the effects on memory and depression in ovariectomized mice. Of the three cultivars, the black maca variety showed the best activity [44]. 

*L. peruvianum* is rich in compounds called glucosinolates. It has been proven that glucosinolates have the ability to inhibit AChE in a dose-dependent manner. Blazevic et al. demonstrated that these secondary metabolites from *Bunias erucago* extracts, like gluconapin, glucoerucin, and glucoraphanin, inhibited the AChE enzyme by about 53% [45], whereas glucosinalbin did so by about 40.9% [25]. Recent studies also confirm that the products of glucosinolate hydrolysis, isothiocyanates, have AChE-inhibiting properties. Phenyl derivatives of isothiocyanates show promising AChE-inhibitory properties [24].

In the presented work, we used a standard method evaluating the AChE activity of newly characterized glucosinolates present in maca. Due to the use of unified methodology, we are able to make direct comparisons with previously characterized compounds. We must emphasize that the inhibitory activity of glucosinolates was within one order of magnitude (at a similar level) of a number of efficient cholinesterase inhibitors—terpenes and phenylpropanoids—previously studied by us in the very same manner [46]. Moreover, glucosinolates exerted much higher inhibitory activity than physodic acid isolated from *Hypogymnia physodes*, characterized in one of our recent works [47]. Although the cholinesterase-inhibiting activity of glucosinolates is lower than the activity of very efficient inhibitors—flavonoids (e.g., quercetin, rutin, or kaempferol) recently identified in selected fruits [48])—this activity can be judged applicable.

The results presented herein underline that next to the colour of Peruvian maca phenotype, the tuber size is an important factor in terms of glucosinolate content. This observation is crucial, as so far there is no specific method to classify tubers according to their size, and the cultivated tubers from a given plantation are collected together. The obtained results draw attention to the fact that in the context of glucosinolate content and possible preventive use of the tubers in memory impairment conditions, the important issue remains proper crop collection.

### 2.5. Modelling

**Figure 6:**
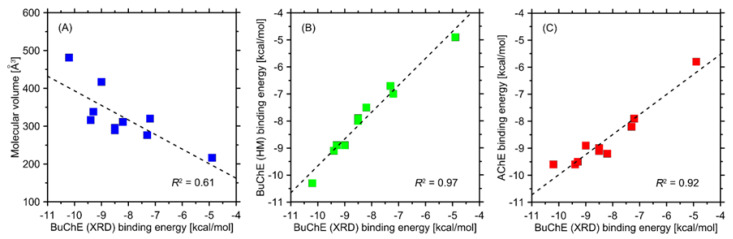
(**A**) Correlation between the average binding energies determined for X-ray crystal structure (XRD) of BuChE and the molecular volume of studied compounds. (**B**) Correlation between binding energies obtained for X-ray crystal structure (XRD) of human BuChE and homology model (HM) of equine BuChE. (**C**) Correlation of binding energies determined for AChE and BuChE and the same set of ligands. Determination coefficients are given as well.

The ligand–protein-binding energies found during docking simulations are given in Table 4 and graphically illustrated in Figure 6. Apart from compound 1-isothiocyanato-9-methanesulfinylnonane, which displays significantly distinct structure, the rest of compounds exhibit relatively similar magnitudes of binding energies. Independently of the considered protein, the strongest binding is always exhibited by compound **G9,** whereas the weakest is exhibited by 1-isothiocyanato-9-methanesulfinylnonane. All negative energy values clearly speak for favorable binding in all considered cases.

A series of interesting correlations is revealed when looking closer at calculated binding energies (Figure 6). Firstly, the binding energies are fairly well but negatively correlated with the molecular sizes of docked ligands (expressed here as molecular volume). This suggests that the increased size of the -R substituent (see Figure 6) positively influences the binding strength and, therefore, inhibition potency. Secondly, the binding energies obtained for a series of X-ray crystal structures of human BuChE are strongly correlated with analogous values for equine BuChE structure obtained from homology modeling. This result is expected due to high sequence identity between these two proteins. Nevertheless, it also speaks for the correctness of the homology model. Finally, a good correlation is observed between binding energies obtained for AChE and BuChE. This also is expected due to the close nature of both enzymes and structural similarities in their binding sites. It is worth noting that in the last two cases, except for a good correlation, there exist only minor deviations in absolute binding energies determined for the same ligand and a given pair of proteins (on average, 0.35 and 0.57 kcal/mol, respectively).

**Figure 7:**
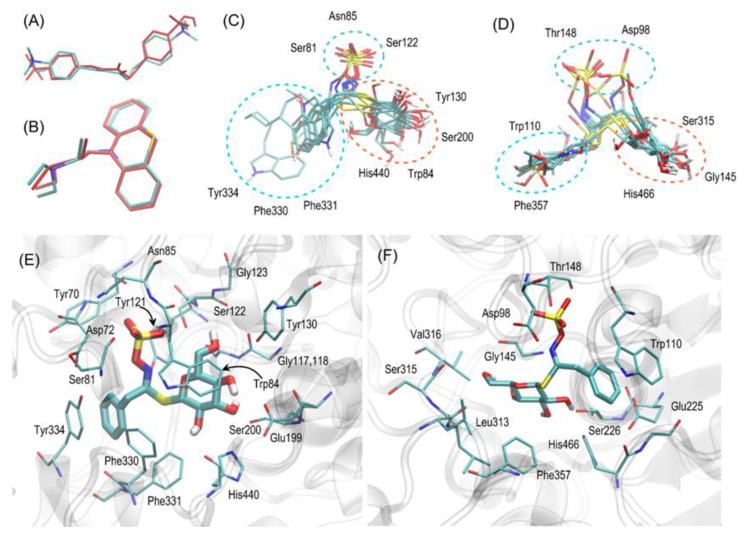
(**A**,**B**) The graphical illustration of the validation results, i.e., the superposition of the most favorable ligand poses found during docking (colored in red) with the position of the co-crystalized ligand, present in the binding cavity of AChE (**A**) or BuChE (**B**) (colored by atom type). (**C**,**D**) The superposition of the most favorable poses of all ligands interacting with either AChE (**C**) or BuChE (**D**). The selected amino acids creating the most essential ligand–protein contacts are given separately for each of ligand moieties. (**E**,**F**) The most favorable location of compound glucotropaeolin molecule bound to either the AChE (**E**) or BuChE (**F**) structure. The ligand molecule is shown as thick sticks, whereas all the closest amino acid residues (of distances no larger than 0.4 nm) are represented by thin sticks. The description of the interaction types is given in the text.

The results of the docking studies have been analysed with respect to the mechanistic interaction pattern that may be significant in the context of interpretation of the obtained binding energies. The summary given below relies on analyzing the ligand–protein contacts that take place if the distance between any corresponding atom pair is smaller than the arbitrarily accepted value of 0.4 nm. Figure 7 shows the superposition of all most favorable ligand poses with the most characteristic ligand-protein interactions. Let us remark that compound 1-isothiocyanato-9-methanesulfinylnonane, sharing little structural similarity with remaining compounds, was excluded from the discussion below. 

All ligands prefer roughly the same binding position in the enzyme cavity, which enables them to block catalytic sites (proximity to the tryptophan and histidine residues). The relative molecular orientation is (in majority of the cases) the same among compounds interacting with the same protein. 

In the case of AChE, the –R substituent (usually of either aromatic or aliphatic character) interacts with the cluster of aromatic amino acid sidechains (Phe330, Phe331, and Tyr334). The corresponding interactions are of either p-p, CH-p stacking or simply hydrophobic nature. The opposite site of the ligand molecule (glucopyranose moiety) interacts with hydrogen-bonding-acceptor-containing amino acid residues (Glu199, Ser200, Tyr130, and His440) mainly by hydrogen bonding. Additionally, the CH-p stacking involving Trp84 and hydrophobic patch in the glucose ring is observed (This type of interaction is characteristic of non-charged carbohydrate–protein binding [49]. As indicated by the dispersion of the poses (including secondary and tertiary ones), the glucopyranosidic moiety is much more conformationally flexible in comparison to the –R substituent. Finally, the charged sulfate group, located in the central part of the ligand molecule, creates some attractive, hydrogen-bonding-mediated interactions with Ser122 and Asn85 (backbone fragment). As the sulfate moiety is located close to the entrance to the binding cavity, it is also possible that multiple interactions with polar water molecules are possible (as no explicit solvent was considered during the docking procedure, this has not been definitively confirmed). 

In the case of BuChE, an important reorientation of the ligand in the binding cavity may be observed in comparison to AChE. Namely, the –R substituent is shifted toward Trp110 (structural analogue of Trp84 in Ache), interacting with it through p-p stacking. P-p interactions are possible also in the case of Phe357 but are probably significantly less intense due to increased R-Phe357 distance. At the same time, the glucose moiety exhibits hydrogen bonding with backbone fragments of Ser315, Gly145, and the side chain of His466 (structural analogue of His440 in AChE). Thus, the whole ligand molecule is slightly shifted toward the more polar edge of the binding cavity. The sulfate group is again located closest to the binding cavity entry and, thus, probably interacts with water molecules. Additionally, its hydrogen binding with Thr148 and Asp98 (backbone) were identified. 

The observed differences between most favorable orientations of the docked ligands interacting either with AChE or BuChE can be ascribed to the diverse composition of the corresponding binding cavities. The cavity of BuChE lacks the cluster of aromatic residues Tyr-Phe-Phe which was responsible for favorable interactions with –R in the case of AChE. Therefore, reoriented –R moiety seeks attractive interactions with Trp110, whereas the glucose group can still interact with Ser226 and His466. 

Finally, let us note that in the case of some compounds and—especially in secondary, tertiary, or quaternary poses—systematic reorientations of the ligand in the binding cavity were observed. Namely, the position of the –R substituent and glucopyranosidic group are swapped. In other words, AChE-characteristic poses become close to the BuChE ones and vice versa. Such an alternative interaction pattern is reasonable to accept, as the carbohydrate moiety can exhibit attractive interactions with either Trp or Phe/Tyr moieties. The same can be said about the aromatic –R substituent. Although the present docking study classifies such binding modes as only secondary ones, the accompanying binding energies are close to the most favorable values (difference by at most 1 kcal/mol). Thus, it cannot be excluded that in real systems, when the solvent and temperature effects are present, the ligand position can be a superposition of those two alternative orientations.

## 3. Materials and Methods

### 3.1. Plant Material and Extraction Conditions

Plant material studied in this paper represents dried tubers of Maca (*Lepidium peruvianum* syn. *L. meyenii)* collected in the highlands of the Peruvian Andes in the Junín plateau (2017 and 2019 harvesting seasons) and Ancash cultivation area (2017 harvest) as summarised in Table 5.

Maca cultivated in the Junín plateau was harvested on two occasions. Sampling J-17 was conducted on 1 October 2017 from plantation in Tarma Province, Tapo District, some 30 km west of Tarma city at 4174 m above sea level (a.s.l. ±20 m). Tubers were sampled from an area of approximately 250 m radius surrounding the geolocation coordinates 11°24′12″S, 75°35′48″W according to GPS reading. 

**Figure 8:**
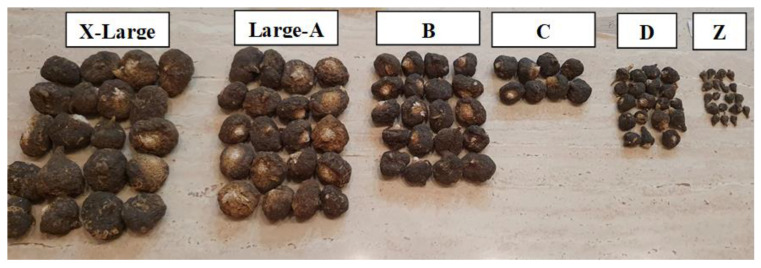
An example of distribution of black maca tubers allocated to size classes dependent on physical size and weight in the black phenotype of *L. peruvianum synon. L. meyenii*).

Maca tubers from the 2019 sampling in Junín, (J-19), originate from the cultivation site on the northeast-facing slopes of the Andes and were harvested on 8 October 2019 at an elevation of 4156 m a.s.l. (±25 m) in an area of approximately 300–350 m radius surrounding the GPS location point 11°06′23″S 75°58′47″W. All traditionally harvested maca tubers of mixed phenotypes harvested in the Junín plateau are from samplings J-A and J-B were transported to a drying location site in Tarma (3148 m a.s.l.) at 11°25′19″ S 75°41′47″ W, where tubers were traditionally dried, separated into individual phenotypes black, red, purple and yellow, and selected into different sizes within individual phenotypes as per the example given in Figure 8. Selected fresh maca tubers from the J-19 location were chopped and juice was squeezed out of the crushed tubers for use in analyses. Separated fresh pulp was discarded. 

Maca tubers cultivated in the Ancash region (A-17) were collected in mid-October 2017 in Ancash, Huanuco in Margos, Margos District, 105 km by car southeast of Huallanca from the cultivation area at 4242 m a. s. l. (±35 m) of an approximately 250 m radius facing the northeast slope of a hill surrounding the GPS point 9°46′8″ S, 76°59′06″ W. Harvested maca tubers were traditionally sun-dried at a plantation site in Ancash, transported for selecting phenotypes and tuber sizes in Huallanca storage, delivered to a processing plant in Lima to be pulverised and packaged, then sent by courier to Lublin laboratory, the Department of Pharmacognosy with Medicinal Plants Garden. The voucher specimen of every sample is kept by the corresponding author in the Department of Pharmacognosy with Medicinal Plants Garden, Medical University of Lublin. All original specimens used in the study were authenticated by Professor Henry O. Meissner directly after collection.

All *L. peruvianum* extracts used in the study were obtained with 50% aqueous ethanol [37] (*v*/*v*) by ultrasound-assisted extraction technique. Three extraction cycles of 30 min each were performed on 2 g of the same plant material using fresh portions of extracting solvent at room temperature. The selected solvent to solid ratio was 1:1 (*v/v*). After extraction, the extracts were joined and evaporated to dryness using a vacuum evaporator at 50 °C. The obtained residue was weighed and stored in a refrigerator at 5 °C until the analysis. Juice samples were produced by cutting fresh bulbs of *L. peruvianum* and placing them into a slow juicer. The collected samples were later centrifuged, filtered through a syringe filter (nominal pore size of 0.2 µm), and used for chromatographic analysis.

### 3.2. Reagents

Ethanol at 96% concentration was purchased from Avantor Performance Materials (Gliwice, Poland) for extraction. HPLC-MS purity water, acetonitrile and formic acid were obtained from J.T. Baker (Phillipsburg, NJ, USA). The reagents used in the AChE and BuChE inhibition assays, namely acetylthiocholine iodide (ATChI), butyrylcholine iodide (BuTChI), 5,5′-Dithiobis (2-nitrobenzoic acid) (DTNB), Tris buffer, sodium chloride (NaCl), and magnesium chloride hydrate (MgCl_2_·6H_2_O), were provided by Sigma Aldrich (St. Louis, MO, USA). The standard of glucotropaeolin (purity > 98%) used to plot the calibration curve was purchased from Extrasynthese (Genay, France). For bioactivity studies, the following standards of glucosinolates were obtained at purities exceeding 95%: glucotropaeolin (**G2**), glucolimnanthin (**G3**), methoxyglucobrassicin (**G4**), and glucosinalbin (**G1**).

### 3.3. Qualitative and Quantitative Analysis of the Extracts by High Performance Liquid Chromatography Coupled with Mass Spectrometry (HPLC-ESI-QTOF-MS/MS)

The HPLC-ESI-QTOF-MS/MS platform from Agilent Technologies (Santa Clara, California, USA) was used to analyse the composition of *L. peruvianum* extracts. For the separation process, an HPLC chromatograph (1200 series) equipped with a Zorbax Eclipse Plus RP-18 chromatography column (150 mm × 2.1 mm; dp = 3.5 µm), a degasser (G1322A), a binary pump (G1312C), an autosampler (G1329B), a photodiode array detector (G1315D), and a mass spectrometer (G6530B) were used. Agilent MassHunter Workstation Software (version B.10.00) was used to acquire the MS spectra and process the data.

The temperature of the HPLC thermostat was set at 25 °C and the UV detection wavelengths at 254, 280, 320, and 365 nm. The operating wavelength range of UV-Vis/DAD detector was 190–600 nm. Chromatographic separation was performed using a 10 µL injection volume with a flow rate of 0.2 mL/min in a 45 min gradient elution program. Mobile phases consisted of eluent A (0.1% formic acid in water, *v*/*v*) and eluent B (acetonitrile solution with 0.1% formic acid added). The gradient elution was as follows: 0 min—1% of B, 4 min—20% of B, 20 min—60% B, 35–38 min—95% B, 39–45 min—1% of B.

The mass spectrometer measurements were carried out under the following conditions: gas temperature and shield gas temperature were 350 and 325 °C, respectively; gas flows were set at 12 L/min; capillary voltage at 3500 V; fragmentor voltage at 120 V; collision energies at 10 and 20 V; skimmer voltage at 65 V; nebulizer pressure at 35 psig. The collected spectra were scanned in the m/z 100–1700 Da range in the negative ionization mode that visualizes glucosinolates, as well as in the positive ionization mode. Two of the most intense signals seen in the TIC spectrum were automatically fragmented to obtain MS/MS spectra. After collecting two spectra for a given m/z value, the selected signals were excluded for 0.2 min from further fragmentation.

### 3.4. Statistical Analysis of Data

#### 3.4.1. Chemometric Analyses

All the chemometric analyses and visualizations were performed using R v4.1.2 [50] programming language in RStudio [51] software with pracma [52], factoextra [53], matlib [54], and corrplot [55] packages installed. 

#### 3.4.2. Absolute (**ABS**) Approach vs. Relative (**REL**) Approach

In the **ABS** approach, nine variables (nine compounds) representing the absolute detector responses, i.e., peak areas for a given compound, were autoscaled in order to enable standard procedures of exploratory analysis, i.e., principal component analysis (**PCA**) and hierarchical clustering (**HC**) (Table S2 in the Supplementary File). In the **REL** approach, all peak areas for nine traced compounds of every given sample were summed to 100%, hence the amount of every compound was represented as a percentage share of a total detector response of a given sample (Table S5 in the Supplementary File). After such a percentage rescaling, all the variables were also autoscaled in a similar fashion to the **ABS** approach.

#### 3.4.3. Principal Component Analysis (**PCA**)

After the standard, formal decomposition of the covariance matrices were calculated for the **ABS** (absolute) and **REL** (relative) autoscaled datasets, three principal components (**PCs**) were first considered relevant in both cases. After the selection of the relevant **PCs**, their vectors were rotated in space in order to maximize the values of correlation coefficients between the original variables and the three orthogonal factors using the VARIMAX algorithm. In every case, samples scores in the space of the resulting varivectors (dimensions) were calculated by multiplying the matrix of the original variables’ loadings in the space of varivectors by the matrix of the autoscaled **ABS**/**REL** dataset.

#### 3.4.4. Correlation Analysis

Matrices of the coefficients of linear correlation (**R**) were prepared using Pearson’s definition of linear correlation.

#### 3.4.5. Hierarchical Clustering (**HC**)

Hierarchical clustering (**HC**) for the **ABS** and **REL** approaches was performed using the Ward method based on a standard matrix of Euclidean distances between the samples.

### 3.5. Quantitative Analysis of Glucotropaeolin in the Analysed Extracts by HPLC-ESI-QTOF-MS/MS

Glucotropaeolin, as the most abundant glucosinolate in the tested extracts, was selected for the quantitative analysis to present the differences in its content between the tested extracts. The quantitative results were prepared for the standard compound (purity >98%, Extrasynthese, Genay, France) on the basis of its calibration curve, plotted for 7 different concentrations within the range of 0.0039–0.25 mg/mL that were obtained by diluting the stock solution with a concentration of 1 mg/mL. The same volumes (10 µL) of every solution were injected on the column and developed in the same above-described chromatographic method as for the qualitative assessment. The linearity range was observed for the concentration range of glucotropaeolin from 0.0039–0.0625 mg/mL. For these concentrations, the calculated calibration curve equation was y = 1,970,017,678x + 2,232,216 and the coefficient of determination (R^2^) was equal to 0.99084.

The dried extracts from different varieties of *L. peruvianum* were redissolved in 50% ethanol (*v*/*v*) to the final concentration of 5 mg/mL, filtered through nylon syringe filters (nominal pore size 0.22 um), and subjected to chromatographic analysis in triplicate.

### 3.6. AChE- and BuChE Inhibition Assay

The selected total extracts (BL_A-J, BL_B-J, BL_C-J, BL_D-J, BL_Z-J, BL_B-J, RE_B-J, GR_B-J, YE_B-J) dissolved in DMSO at a concentration of 10 mg/mL and reference compounds (GL, GTP, GB, GS) at a concentration of 1 mg/mL. These samples were tested for their BuChE- and AChE-inhibitory activity according to the previously published protocol described below. 

The assay was essentially performed as described by Szwajgier et al. (2021) [56]. AChE and BuChE were at a concentration of 0.28 U/mL, ATChI and BuTChI were at a concentration of 1.5 mmol/L, and DTNB (0.3 mmol/L) contained 11.89 mg DTNB, 58.44 mg NaCl, and 40.66 mg MgCl_2_·6H_2_O, all per 100 mL of Tris buffer. The measurement of the absorbance at 405 nm was performed using a GloMax^®^ Explorer GM3500 plate reader (Promega, Madison, WI, USA) using the Software version 3.2.3. All samples were tested in triplicate. Three blank tests were also performed together with one false positive control for every samples. In the first blank test, the sample was replaced with 10 µL DMSO, the second contained 10 µL Tris instead of the sample; in the third, the enzyme and the sample were replaced with Tris buffer and “false-positive” blank sample according to Rhee et al. [57].

### 3.7. Molecular Docking of Glucosinolates to AChE and BuChE

The ligand molecules were drawn by using Avogadro 1.1.1 [58] and initially optimized within the UFF force field [59] (5000 steps, steepest descent algorithm). Flexible and optimized ligand molecules were docked into the binding pocket of the protein structures either found in the PDB database or prepared by using a homology modeling procedure. In the case of AChE, the 1EEA PDB entry (AChE from electric eel, X-ray resolution: 0.45 nm) was used. In the case of BuChE, no equine BuChE structure was deposited in PDB. Therefore, seven different human BuChE structures (PDB: 6EQP, 4BDS, 1XLW, 4XII, 6ESJ, 6ESY, and 6QAC of resolutions equal to 0.235, 0.210, 0.210, 0.270, 0.298, 0.280, and 0.277 nm, respectively) and, additionally, a homology model prepared on the basis of the highest resolution 4BDS structure, were used. Note that the equine BuChE has ~90% amino acid similarity with the human sequence [60] and nearly all differences are located outside binding cavity. The homology model was prepared by using the SWISS-MODEL server [61,62] and all inherent, default procedures. 

Docking simulations were carried out in the AutoDock Vina software [63]. The procedure was performed within the cubic region of dimensions of 18 × 18 × 18 Å^3^, which covers all the originally cocrystallized ligands present in the considered PDB structures as well as the closest amino acid residues which exhibit contact with those ligands. All the default procedures and algorithms implemented in AutoDock Vina were applied during the docking procedure. The torsional angles in ligand molecules as well as the selected amino acid sidechains within the binding cavities of both proteins were allowed to rotate. In the case of human BuChE, the predicted binding energies were averaged over all seven protein structures. The visual inspections of each pose of the docked ligand (after aligning with respect to the given protein structure) were carried out in order to assure that the partial energies contributing the final, averaged, binding energy value, correspond to the structurally analogous orientations. Only the lowest energy values, corresponding to the most energetically favorable complexes, were considered during subsequent analysis. The procedure was initially validated by docking the ligands cocrystalized with proteins and present in the 6EQP and 1E3Q (AChE from *Torpedo californica*). The graphical illustration of the validation results is given in Figure 7.

## 4. Conclusions

The results presented above provide evidence of the existence of substantial antienzymatic activity of several well-identified maca compounds against AChE and BuChE, thus confirming that *L. peruvianum* is a rich source of active glucosinolates, thus showing promise for possible application of selected maca phenotypes in developing therapeutic products for treating medical conditions related to memory impairment. It was proved that the potential of the plant is related both to the phenotype (color) and to the size of Peruvian maca tubers.

## Figures and Tables

**Table 1:** The composition of the 50% EtOH (*v*/*v*) extract from tubers of black A variety of *Lepidium peruvianum* recorded in the negative ionization mode. (RDB—rings and double bond number, delta—error of mass measurement, ion—ionization mode: positive or negative, ND—not detected). Compounds depicted in bold were subjects of chemometric considerations.

No.	Ion. (+/−)	Rt (min)	Molecular Formula	*m*/*z* Calculated	*m*/*z* Experimental	Delta (ppm)	RDB	MS/MS	Proposed Compound	References
1	-	1.88	C_6_H_12_O_7_	195.051	195.052	−4.97	1	159, 129	Gluconic acid	[31]
2	-	2.06	C_4_H_6_O_5_	133.0142	133.0142	0.35	2	115	Malic acid	[31]
3	-	2.79	C_6_H_8_O_7_	191.0197	191.0209	−6.11	3	155, 111	Citric Acid	[31]
4	-	3.09	C_4_H_6_O_4_	117.0199	117.0193	−4.81	2		Succinic acid	[32]
**5**	**-**	**5.48**	**C_14_H_19_NO_10_S_2_**	**424.0378**	**424.0387**	**−2.21**	**6**	**274, 182**	**Glucosinalbin (G1)**	[33]
**6**	**-**	**9.26**	**C_14_H_19_NO_9_S_2_**	**408.0428**	**408.0430**	**−0.38**	**6**	**258, 195**	**Glucotropaeolin (G2)**	[33]
**7**	**-**	**12.4**	**C_15_H_21_NO_10_S_2_**	**438.0534**	**438.0541**	**−1.57**	**6**	**196**	**Glucolimnanthin (G3)**	[33]
**8**	**-**	**13.3**	** C_17_H_22_N_2_O_10_S_2_**	**477.0643**	**477.0665**	**−4.58**	**8**		**4-methoxyglucobrassicacin (G4)**	[33]
9	-	15.43	C_11_H_18_N_2_O_4_	241.1194	241.1199	−2.15	4	197	Pyroglutamylleucine	[34]
**10**	**-**	**15.71**	**C_22_H_36_ON_2_S**	**551.1727**	**551.1754**	**−2.79**	**6**	**389, 235**	**4-methoxyindolyl-3-hexylhydroxyglucosinolate (G5)**	[35]
**11**	**-**	**15.8**	**C_11_H_21_NOS_2_**	**246.0992**	**246.1006**	**−5.75**	**2**	**210, 130**	**1-isothiocyanato-9-methanesulfinylnonane (G6)**	[36]
12	-	16.8	C_15_H_29_O_10_NS_2_	446.1154	446.1116	9.87	2	ND	Indolyl-5-methylglucosinolate (IMG)	[37]
**13**	**-**	**17.02**	**C_12_H_24_O_7_N_2_S_2_**	**371.0941**	**371.1000**	**−9.9**	**2**	**249, 121**	**Pent-4-enylglucosinolate (G7)**	[33]
**14**	**-**	**17.6**	**C_13_H_25_NO_10_S_3_**	**450.0568**	**450.0570**	**−0.48**	**2**	**316**	**Glucoalyssin (G8)**	[33]
**15**	**-**	**17.7**	**C_22_H_38_O_9_N_2_S_2_**	**521.1985**	**521.2016**	**−3.67**	**5**	**ND**	**indolyl3-hexyl-4-methyl-cyclohexaneglucosinolate (G9)**	[33]
16	-	20.3	C_11_H_10_O_6_	237.0405	237.0406	−0.58	7	121	Malic acid benzoate	[16]
17	-	20.35	C_7_H_6_0_2_	121.03	121.0295	−4.07	5	ND	Benzoic acid derivative	[33]
18	-	20.35	C_24_H_18_O_12_	497.0725	497.0697	5.72	16	381, 237	Fucodiphlorethol	[38]
19	-	25.6	C_18_H_32_O_5_	327.2177	327.2195	−5.49	3	229, 171	Trihydroxy-(3)-octadecadienoic acid	[39]
20	-	26.9	C_18_H_34_O_5_	329.2333	329.2363	−8.94	2	211, 171	Pinellic acid (9(S),12(S),13(S)-Trihydroxy-10(E)-octadecenoic acid)	[40]

**Table 2:** The results of the quantitative determination of glucotropaeolin in the studied extracts (green color corresponds with the highest quantity of G2 in the extracts, wheras red colour shows the samples with the lowest calculated content).

No	Sample Code	Percentage Content	SD	No	Sample Code	Percentage Content	SD
1	BL_A-J	1.514	0.17	20	GR_C-J	1.513	0.10
2	BL_B-J	0.954	0.08	21	GR_D-J	1.301	0.12
3	BL_C-J	0.632	0.05	22	GR_X-J	0.540	0.05
4	BL_D-J	0.875	0.10	23	GR_Z-J	1.362	0.16
5	BL_X-J	0.270	0.02	24	GR_JU	ND	0.00
6	BL_Z-J	0.337	0.03	25	PU_A-J	0.214	0.03
7	BL_JU	ND	0.00	26	PU_B1-J	0.078	0.01
8	CH	0.081	0.01	27	PU_B2-J	0.239	0.02
9	BL_gel-AN	0.624	0.08	28	PU_C-J	1.572	0.16
10	MIX_gel-AN	ND	0.00	29	PU_D-J	1.155	0.09
11	BL_Bp-AN	0.965	0.11	30	PU_JU	ND	0.00
12	RE_Bp-J	0.923	0.09	31	YE_A-J	0.749	0.07
13	RE_LA-J	0.344	0.02	32	YE_B-J	0.850	0.09
14	RE_LAgel	0.061	0.01	33	YE_B2	0.850	0.06
15	RE_gel_AN	0.103	0.01	34	YE_D-J	0.474	0.03
16	RE_SM	0.242	0.02	35	YE_JU	ND	0.00
17	GR_A-J	0.518	0.04	36	YE_Z-J	1.195	0.14
18	GR_B1-J	1.040	0.09	37	YE_B2	0.850	0.06
19	GR_B2-J	0.804	0.06				

**Table 3:** The average results of anti-enzymatic assays for the total extracts and the reference compounds, measured spectrophotometrically in the modified Ellman’s tests.

	BuChE	AChE
% inhibition by the total extracts		
BL_A-J	77.2	24.1
BL_B-J	77.2	22.5
BL_C-J	77.2	22.5
BL_D-J	73.7	19.3
BL_Z-J	72.5	19.3
BL_B-J	77.2	22.5
RE_B-J	71.3	28.9
GR_B-J	76.0	17.0
YE_B-J	75.7	15.3
% inhibition by the reference compounds		
GL	37.05	56.5
GTP	36.2	55.95
GB	39.9	57.6
GS	38.9	56.9
% inhibition by a positive control		
Berberine (0.5 mg/mL)	33.7	-

**Table 4:** The collection of the binding energies during docking study; the calculation results were averaged over all structures available in the same PDB record; the corresponding standard deviations are given.

Compound	Binding Energy (kcal/mol)		
	AChE	BuChE XRD	BuChE Homology Model
**(G2)**	−9.1	−8.5 ± 0.1	−8.0
**(G3)**	−9.2	−8.2 ± 0.3	−7.5
**(G1)**	−9.0	−8.5 ± 0.3	−7.9
**IMG**	−9.6	−9.4 ± 0.3	−9.1
**(G8)**	−7.9	−7.2 ± 0.2	−7.0
**(G4)**	−9.5	−9.3 ± 0.4	−8.9
**(G5)**	−8.9	−9.0 ± 0.2	−8.9
**(G7)**	−8.2	−7.3 ± 0.1	−6.7
**(G9)**	−9.6	−10.2 ± 0.5	−10.3
**G6)**	−5.8	−4.9 ± 0.2	−4.9

**Table 5:** Plant material: coding, description and sampling details prior to delivery to Laboratory Lublin.

No	Sample Code	Sample Description	Specimen Collection Location	Sampling Year	Time of Collection	No	Sample Code	Sample Description	Specimen Collection Location	Sampling Year	Time of Collection
1	BL_A-J	Dried black Large tubers size A (Smaller than X)	Junín (J-19) Maca plantation, Peruvian Andean highlands *	8 October 2019	Between 11am and 2pm local time in Peru	20	GR_C-J	Dried grey tubers size C (smaller than B)	Junín (J-19)	8 October 2019	Between 10am and 2 pm local Peruvian time
2	BL_B -J	Dried black tubers size B (Smaller than A)				21	GR_D-J	Dried grey tubers size D (smaller than C)			
3	BL_C-J	Dried black tubers size C (Smaller than B)				22	GR_X-J	Dried grey tubers size X- Extra Large (larger than A)			
4	BL_D-J	Dried black tubers size D (Smaller than C)				23	GR_Z-J	Dried grey tubers size E (very small–smaller than D)			
5	BL_X-J	Dried black tubers size Extra Large (X)				24	GR_JU	Squeezed juice from fresh grey maca tubers (fresh size A)			
6	BL_Z-J	Dried black tubers size E (smaller than D)				25	PU_A-J	Dried PURPLE maca tubers size A			
7	BL_JU	Pressed juice from fresh black tubers (BL A-J and B-J)				6	PU_B-J	Dried PURPLE maca tubers size B (smaller than A)			
8	CH	Semi-dried dark violet tubers purchased by maca contractor from unknown sources/location origin not known	Tarma (J-17) *	30 September 2017	3 p.m. Local time	27	PU_B2-J	Dried PURPLE maca tubers size B-1-Replicate (smaller than A)			
9	BL_gel-AN	Organic gelatinized BLACK maca tubers, sampled and dried in Ancash and processed in Lima	Ancash (A-17) Maca Plantation, Cordillera Blanca, Peruvian Andes.	Mid-October 2017	Between 10 a.m. and 4 p.m. local Peruvian time	28	PU_C-J	Dried purple maca tubers size C (smaller than B)			
10	MIX_gel-AN	Organic gelatinized tubers of MIXED maca phenotypes				29	PU_D-J	Dried purple tubers size D (smaller than C)			
11	BL_Bp-AN	Organic BLACK tubersopen- air dried on site and pulverised in Lima processing plant				30	PU_X-J	Dried purple tubers size X Extra Large (larger than A)			
12	RE_Bp-J	RED tubers size B dried and powdered-as per above	Junín (J-17)			31	PU_JU	Squeezed juice from fresh purple tubers			
13	RE_LA-J	RED maca large powdered tubers size A				32	YE_A-J	Dried yellow tubers size A			
14	RE_LA gel	RED maca large tubers size A—gelatinised powder				33	YE_B-J	Dried yellow tubers size B (smaller than A)			
15	RE_gel AN	organic gelatinized RED maca powder Maca Plantation Cordillera Blanca, Peruvian Andes	Ancash. (A-17) Cordillera Blanca Peruvian Andes			34	YE_B2-J	Dried yellow tubers size B2—Replicate B (smaller than A)			
16	RE_SM-J	RED maca small tubers –powder	Junín (J-17)			35	YE_D-J	Dried yellow tubers size D (smaller than C)			
17	GR_A-J	Dried grey tubers size A	Junín (J-19)	8 October 2019		36	YE_JU	Squeezed juice from fresh yellow tubers size A			
18	GR_B1-J	Dried grey tubers size B (smaller than A)				37	YE_Z-J	Dried yellow tubers size E (smaller than size D) Extra small			
19	GR_B2-J	Dried grey tubers size B (smaller than A)									

* Tubers from commercial drying yard in Tarma (Junín) on 30 September 2017.

## Data Availability

The supporting data are available the Supplementary File.

## Supplementary Materials

The following supporting information can be downloaded at: https://www.mdpi.com/article/10.3390/ijms23094858/s1.
