# Supplementary material for: The Distribution of Glucosinolates in Different Phenotypes of Lepidium peruvianum and Their Role as Acetyl- and Butyrylcholinesterase Inhibitors—In Silico and In Vitro Studies
Source: Int J Mol Sci. 2022 Apr 27;23(9):4858. doi: 10.3390/ijms23094858 (PMC9101689; doi:10.3390/ijms23094858)
Supplement: Supplementary file 1 [file ijms-23-04858-s001.zip › ijms-1666328-supplementary.pdf]

Supplementary file

The distribution of glucosinolates in different phenotypes of *Lepidium peruvianum* and their role as acetyl- and butyrylcholinesterase inhibitors - *in silico* and *in vitro* studies

Dominik Tarabasz[a], Paweł Szczeblewski[b], Tomasz Laskowski[b], Wojciech Płaziński[c,d], Ewa Baranowska-Wójcik[e], Dominik Szwajgier[e], Wirginia Kukula-Koch[a*+], Henry O. Meissner[f+]

[a]Department of Pharmacognosy With Medicinal Plants Garden, Medical University of Lublin, 1 Chodzki str., 20-093 Lublin, Poland; [b]Department of Pharmaceutical Technology and Biochemistry and BioTechMed Centre, Faculty of Chemistry, Gdańsk University of Technology, Gabriela Narutowicza Str. 11/12, 80-233 Gdańsk, Poland; [c]Jerzy Haber Institute of Catalysis and Surface Chemistry, Polish Academy of Sciences, Niezapominajek 8 , 30-239 Krakow, Poland; [d]Department of Biopharmacy, Faculty of Pharmacy, Medical University of Lublin, Chodźki 4A, 20-093 Lublin, Poland, [e]Department of Biotechnology, Microbiology and Human Nutrition, University of Life Sciences in Lublin, Skromna 8 Street, 20-704 Lublin, Poland; [f]Therapeutic Research, TTD International Pty Ltd., 39 Leopard Ave., Elanora, Gold Coast 4221, Australia; dr.meissner@ttdintnl.com.au

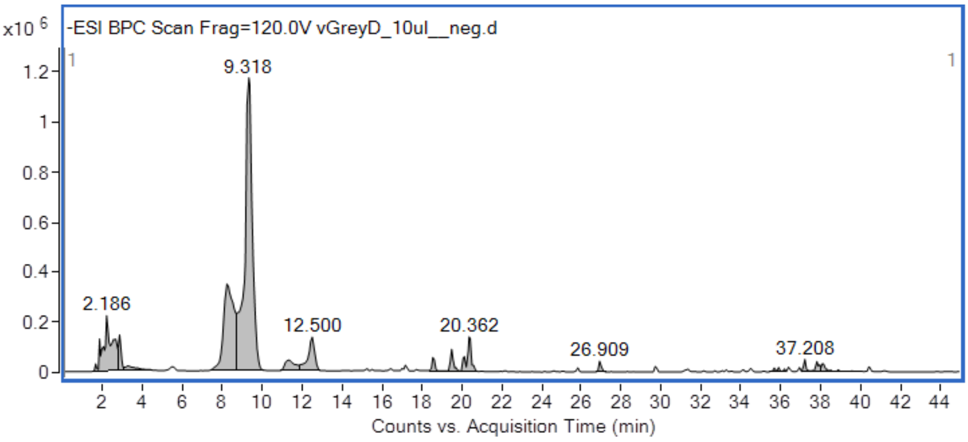

Figure S1. The composition of the 50% EtOH (v/v) extract from black A variety of *Lepidium peruvianum* in the negative ionization mode

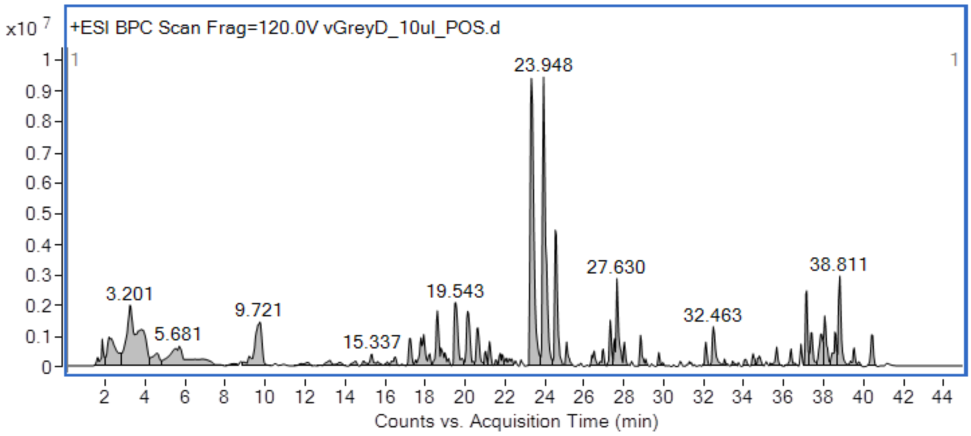

Figure S2. The composition of the 50% EtOH (v/v) extract from black A variety of *Lepidium peruvianum* in the positive ionization mode

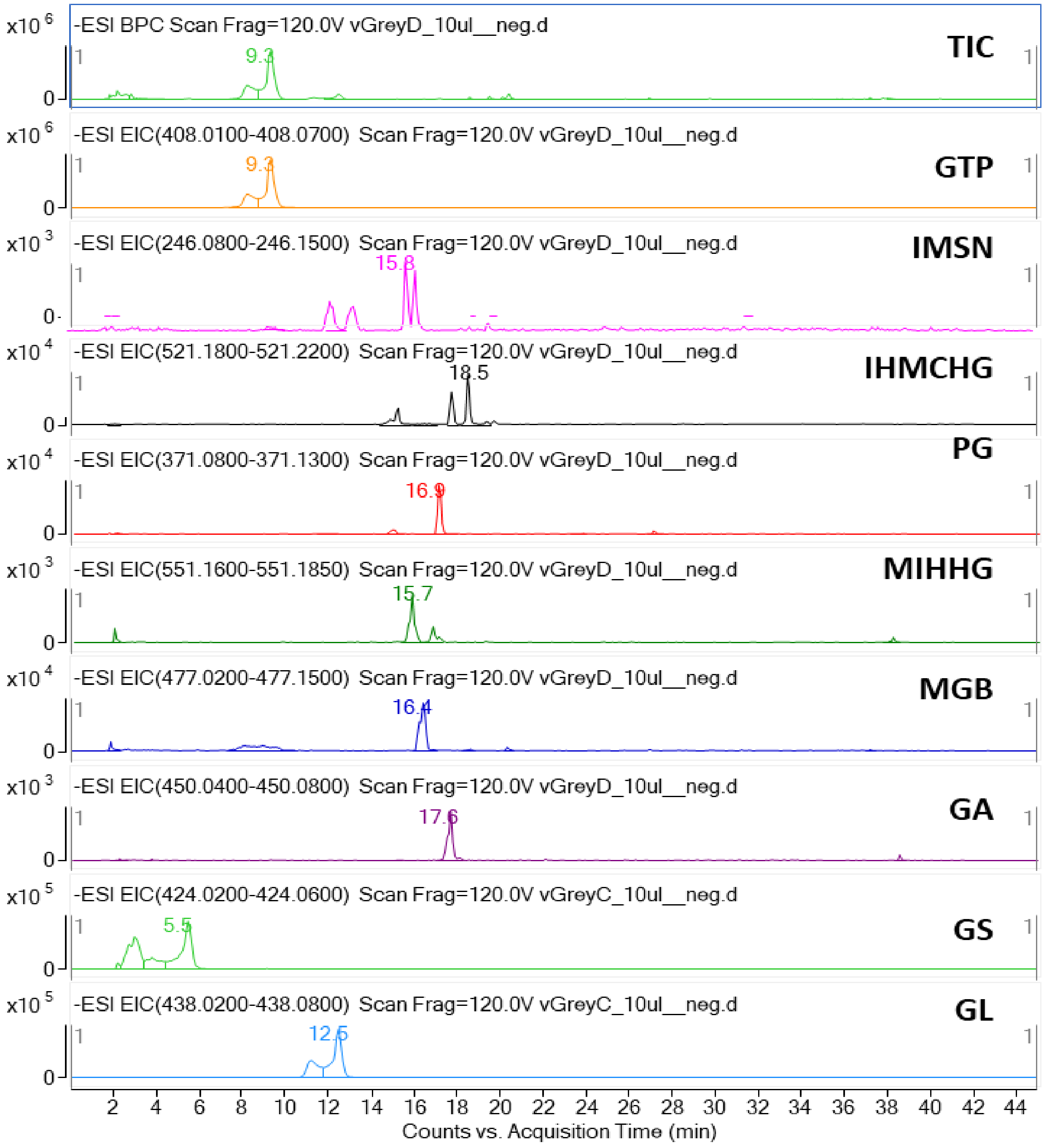

Figure S3. The total ion chromatogram (TIC) recorded in the negative ionization mode for the extract from powdered tubers of grey maca size D together with extracted ion chromatograms (EIC) indicating the presence of glucosinolates (GTP=**G2** - Glucotropaeolin, IMSN=**G6** - 1-isothiocyanato-9-methanesulfinylnonane, IHMCHG=**G9** - indolyl-3-hexyl-4-methyl-cyclohexaneglucosinolate, PG=**G7** - pent-4-enylglucosinolate, MIHHG=**G5** - 4-methoxyindolyl-3-hexylhydroxyglucosinolate, MGB=**G4** - 4-methoxyindolyl-3-methyloxyglucosinolate, GA=**G8** - glucoalyssin, GS=**G1** - glucosinalbin, GL=**G3** - glucolimnantin)

Table S1. The MS/MS spectra of the identified components of the extract visible in the negative ionization mode

MS/MS spectra	Identified compound
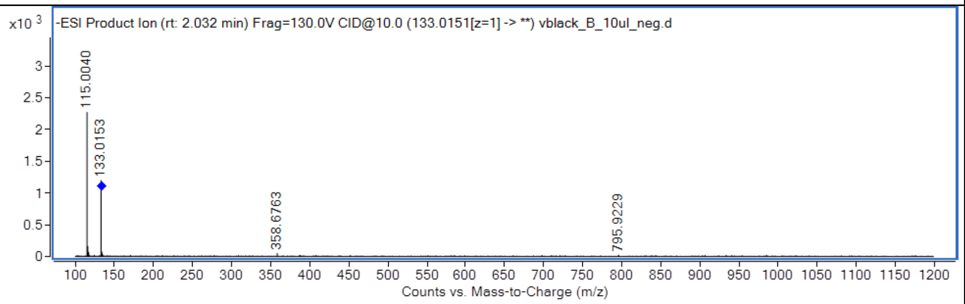	Malic acid
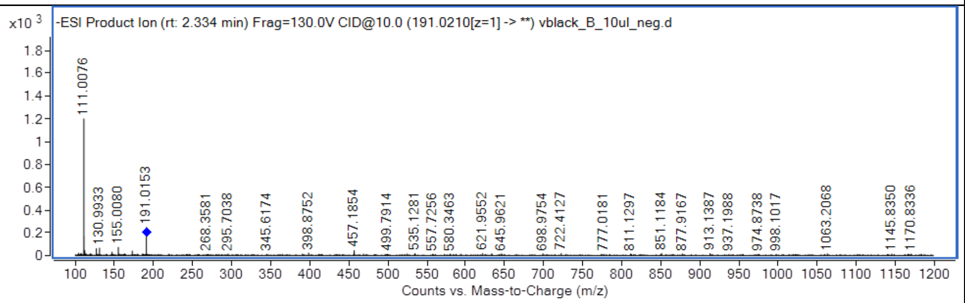	Citric Acid
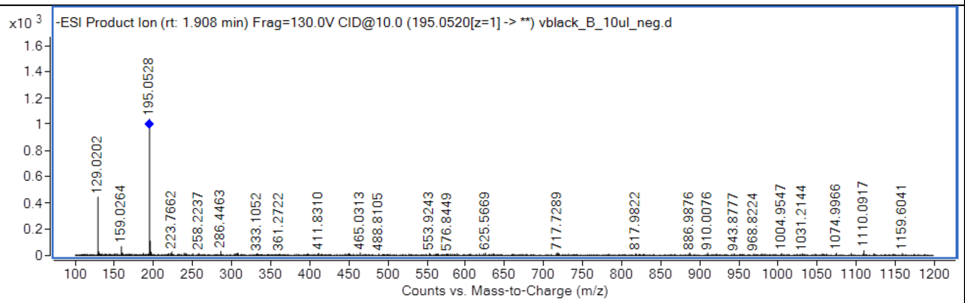	Gluconic acid
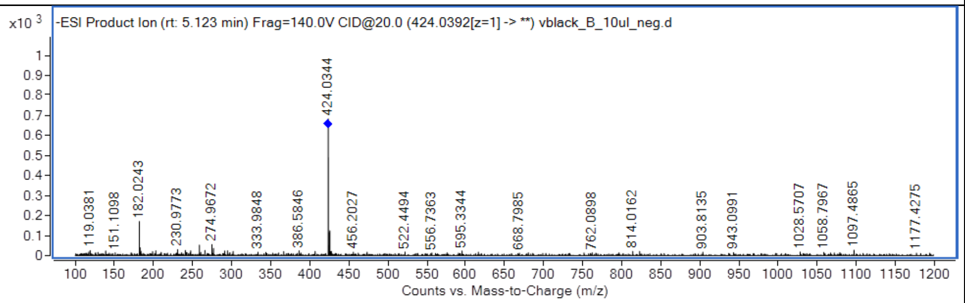	Glucosinalbin

<table>
<tr><td>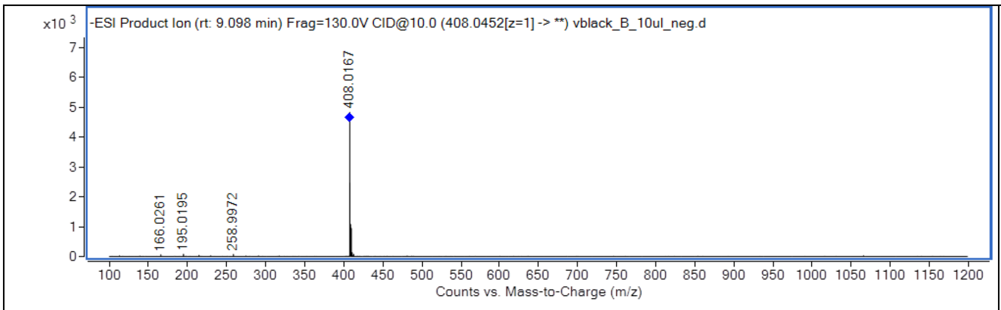
</td><td>Glucotropaeolin</td></tr>
<tr><td>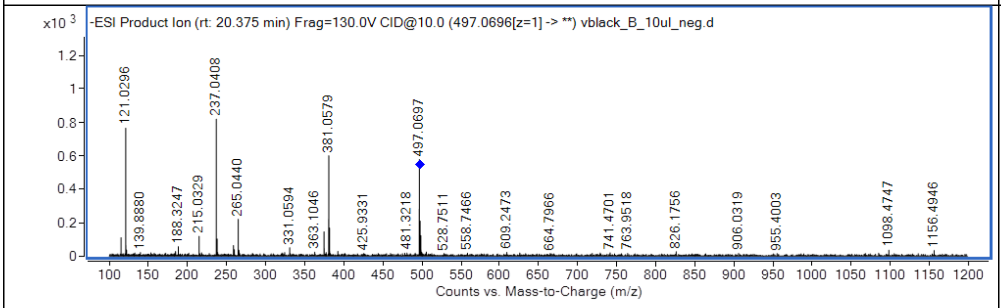
</td><td>Benzoic acid derivative/ Fucodiphlorethol</td></tr>
<tr><td>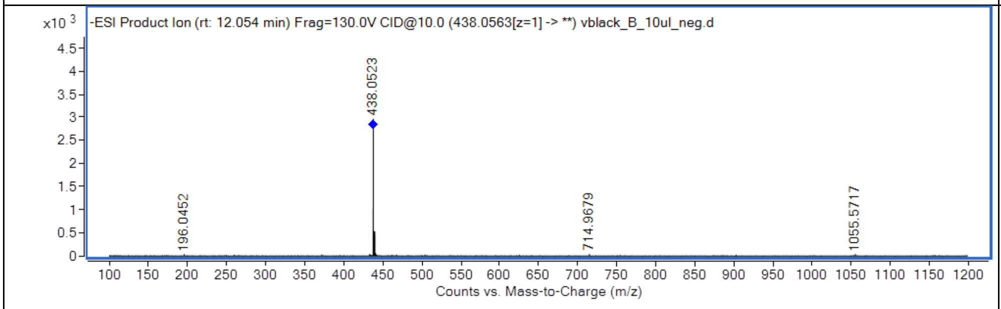
</td><td>Glucolimnathin</td></tr>
<tr><td>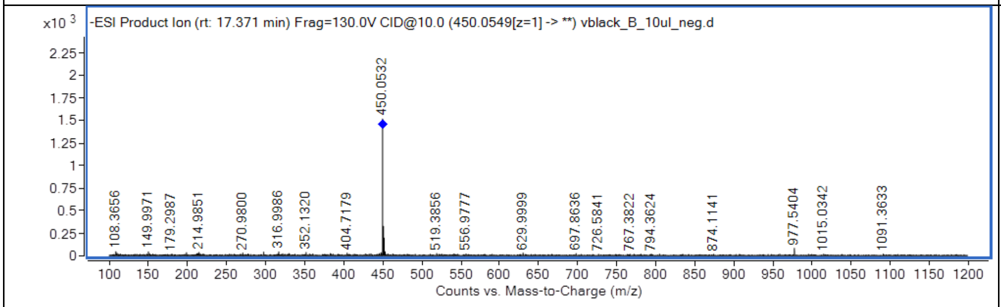
</td><td>Glucoalyssin</td></tr>
<tr><td>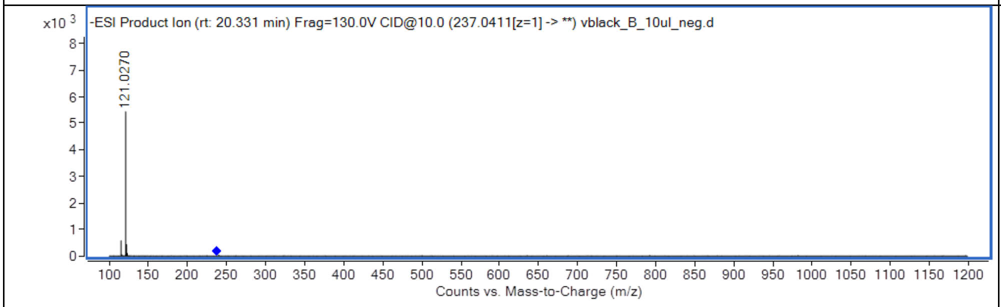
</td><td>Malic acid benzoate</td></tr>
</table>

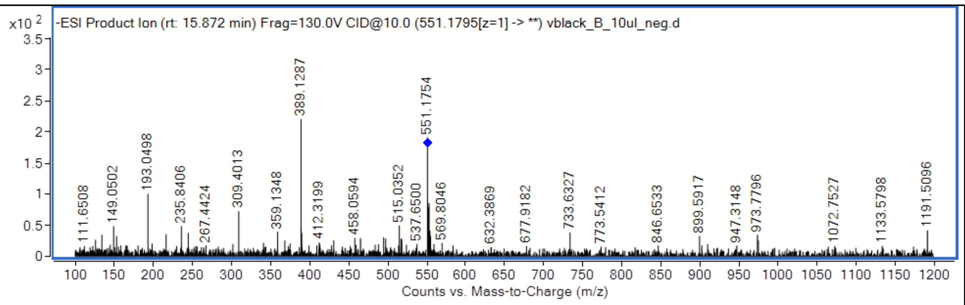

4-methoxyindolyl-3-hexylhydroxyglucosinolate

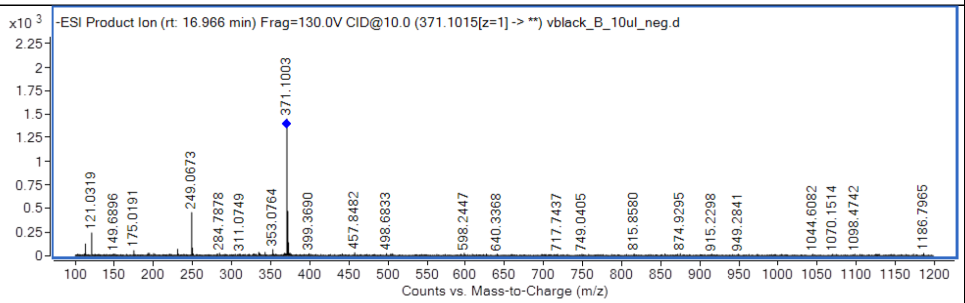

Pent-4-enylglucosinolate

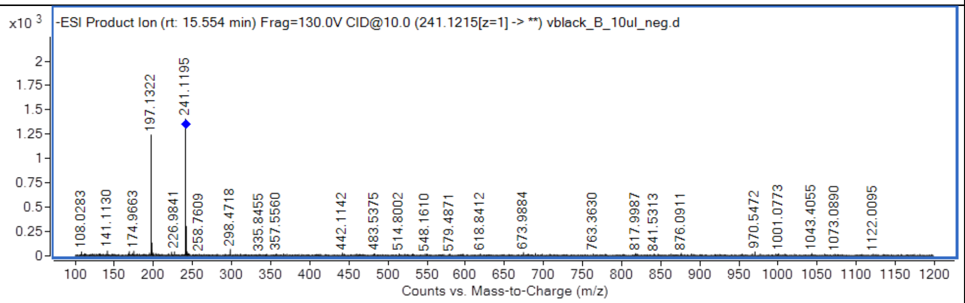

Pyroglutamylleucine

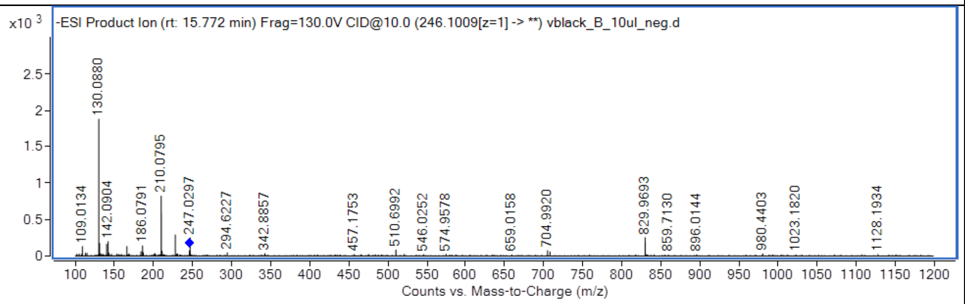

1-isothiocyanato-9-methanesulfinylnonane

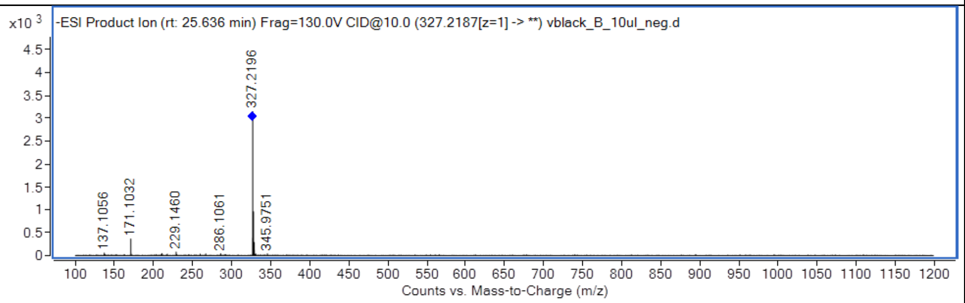

Trihydroxy-octadecadienoic acid

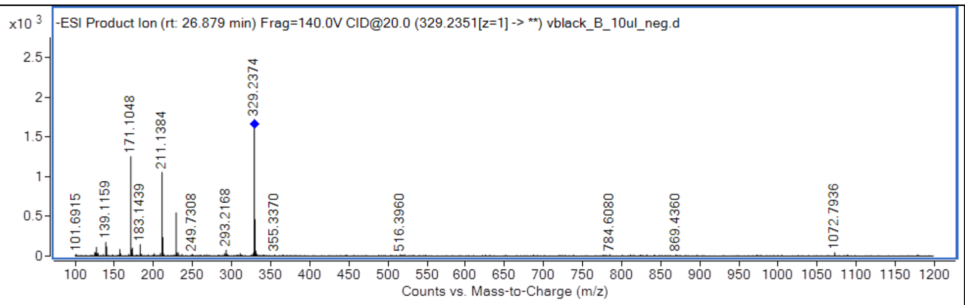	Pinellic acid (9(S),12(S),13(S)-Trihydroxy-10(E)-octadecenoic acid)
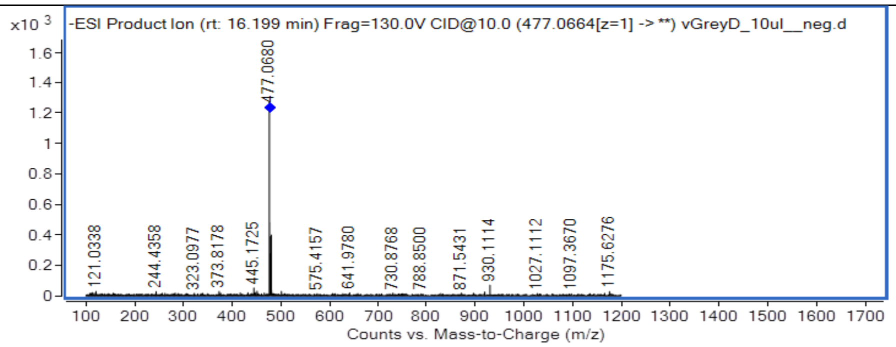	4-methoxyglucobrassicacin

Table S2. The ‘absolute’ approach data table (**ABS**).

Sample code	No.	G2	G6	G9	G7	G5	G4	G8	G1	G3
BL_A	1	2.92E+08	1741743	3586	265387.5	40576	6815.5	943560.5	2434883	32855380
BL_B	2	1.9E+08	1173372	18473	377483	60131	4480	697770	972315.5	20095215
BL_C	3	1.21E+08	1595676	28712	806816.5	116475	34405	312405.5	1476665	13966368
BL_D	4	1.74E+08	1155251	54747	282929.5	105605	43103	473277.5	2283710	16642125
BL_X	5	53850129	984394	6577	240704.5	33288.5	32449.5	121107.5	547056.5	8978421
BL_Z	6	65762830	33448.5	239883	124071	78230	519072.5	48084.5	627747	6723647
CH	7	15716274	1236353	65882	708966	194031.5	0	56395.5	205589	3906229
BL_gel	8	1.09E+08	1588586	21737.5	726558	57375	9915.5	244623.5	1045382	14577931
MIX_gel	9	0	400425	104117	487568	56482.5	5616	4139.5	281205	113469
BL_Bp	10	1.92E+08	871312.5	18646.5	424903	58770	61212.5	415736	1521886	26517616
RE_Bp	11	1.84E+08	261557	46610.5	334317	56864	35883.5	304908	1841570	28773780
RE_LA	12	68575801	302112	25851.5	136213.5	63850.5	18318.5	171619	612997	10755339
RE_LAgel	13	11498005	328208.5	24486.5	346130.5	74161	9340.5	55641.5	101765.5	1959255
RE_gel	14	20509823	1784042	17617	541020	104006.5	0	82320.5	405566.5	3440318
RE_SM	15	48300215	347838	25211.5	384182.5	62380.5	6452.5	98554.5	430109	8400278
GR_A	16	1.03E+08	1365563	10793	343642.5	52080	9585.5	404851	941037	14585134
GR_B1	17	2.02E+08	1344030	12335.5	379528.5	50289.5	34812	628505.5	2042833	28862835
GR_B2	18	1.55E+08	2132562	11595.5	404428.5	54583.5	12044.5	652672.5	1596182	25550725
GR_C	19	2.92E+08	1011538	16022.5	380733.5	68446.5	50739	987584.5	2241915	33895805
GR_D	20	2.54E+08	139570.5	147189	512590.5	219374.5	386324	312464.5	2484093	37314344
GR_X	21	1.27E+08	1151411	29020.5	211754.5	133748	30924	269724.5	1835665	19550278
GR_Z	22	2.67E+08	1133910	127932	335446	363497.5	1056874	360230	2928251	29546764
PU_A	23	42631007	538174.5	150308	266243	314251	14873	103249.5	368624.5	5626706
PU_B1	24	15076461	478001.5	120739	342281.5	337776	23856	65109.5	219084.5	3396035
PU_B2	25	47717650	67074.5	225697.5	150964	205991.5	15030.5	64843	483534.5	11676492
PU_C	26	3.03E+08	1422338	56896	244695	220862	50295	795752	3100773	35375445
PU_D	27	2.2E+08	177048	45092.5	137814	113009.5	0	579161	2401185	37622653
GR_JU	28	0	475671	4359.5	174295	331915	0	0	0	0
PU_JU	29	0	426615	3828	168153	289435.5	0	0	0	0
BL_JU	30	0	130904.5	7094.5	53938.5	470675.5	0	0	0	0
YE_A	31	1.49E+08	656856	26178.5	370031	81799	24137.5	376578.5	1328340	20106477
YE_B	32	1.69E+08	505266.5	108134.5	431920.5	492392	40361.5	387046.5	1266754	21052593
YE_D	33	94599733	530182.5	76089.5	263986	485417.5	40667.5	92946	1124159	14261539
YE_JU	34	0	482571.5	1625	112279	286380	0	0	0	0
YE_Z	35	2.38E+08	401178	89252.5	273368	394113	30222	726126.5	1769694	17970082
YE_B2	36	1.69E+08	505266.5	108134.5	431920.5	492392	40361.5	387046.5	1266754	21052593

Table S3. Eigenvalues and variance explained by the principal components resulting from the **ABS** analysis.

PC	1	2	3	4	5	6	7	8	9
Eigenvalue	3.997722	2.062326	1.119657	0.741948	0.567376	0.320099	0.131897	0.049876	0.009099
% of variance	44.42	22.91	12.44	8.24	6.30	3.56	1.47	0.55	0.10
sum % of variance	44.42	67.33	79.77	88.02	94.32	97.88	99.34	99.90	100.00

Table S4. Loadings of the original variables in the space of the first three varivectors (dimensions) resulting from the **ABS** analysis. One can calculate the coordinates of the samples in space of the first three varivectors by multiplying matrix presented in Table S3 by the autoscaled version of the matrix presented in Table S1.

	**Dim1**	**Dim2**	**Dim3**
G2	0.981	0.100	0.087

G6	0.306	-0.313	0.750
G9	-0.068	0.840	-0.207
G7	-0.034	0.119	0.892
G5	-0.142	0.396	-0.474
G4	0.250	0.805	0.052
G8	0.909	-0.234	0.134
G1	0.949	0.159	0.129
G3	0.956	0.075	0.085

Table S5. The ‘relative’ approach data table (**REL**). Each row sums to 1.

Sample code	No.	G2	G6	G9	G7	G5	G4	G8	G1	G3
BL_A	1	0.884024	0.005275	1.09E-05	0.000804	0.000123	2.06E-05	0.002858	0.007375	0.09951
BL_B	2	0.890427	0.005495	8.65E-05	0.001768	0.000282	2.10E-05	0.003267	0.004553	0.094101
BL_C	3	0.868339	0.011457	0.000206	0.005793	0.000836	0.000247	0.002243	0.010602	0.100277
BL_D	4	0.892366	0.00591	0.00028	0.001447	0.00054	0.00022	0.002421	0.011682	0.085133
BL_X	5	0.831096	0.015193	0.000102	0.003715	0.000514	0.000501	0.001869	0.008443	0.138568
BL_Z	6	0.886805	0.000451	0.003235	0.001673	0.001055	0.007	0.000648	0.008465	0.090668
CH	7	0.711475	0.05597	0.002982	0.032095	0.008784	0	0.002553	0.009307	0.176835
BL_gel	8	0.856703	0.012458	0.00017	0.005698	0.00045	7.78E-05	0.001918	0.008198	0.114325
MIX_gel	9	0	0.275581	0.071655	0.335554	0.038872	0.003865	0.002849	0.193531	0.078092
BL_Bp	10	0.865583	0.003918	8.39E-05	0.001911	0.000264	0.000275	0.00187	0.006844	0.119251
RE_Bp	11	0.853295	0.001212	0.000216	0.001549	0.000264	0.000166	0.001413	0.008535	0.13335
RE_LA	12	0.850161	0.003745	0.00032	0.001689	0.000792	0.000227	0.002128	0.0076	0.133338
RE_LAgel	13	0.798639	0.022797	0.001701	0.024042	0.005151	0.000649	0.003865	0.007069	0.136088
RE_gel	14	0.76288	0.066359	0.000655	0.020124	0.003869	0	0.003062	0.015085	0.127966
RE_SM	15	0.83197	0.005992	0.000434	0.006618	0.001075	0.000111	0.001698	0.007409	0.144695
GR_A	16	0.853698	0.011279	8.91E-05	0.002838	0.00043	7.92E-05	0.003344	0.007773	0.12047
GR_B1	17	0.858464	0.005703	5.23E-05	0.00161	0.000213	0.000148	0.002667	0.008668	0.122473
GR_B2	18	0.836266	0.01148	6.24E-05	0.002177	0.000294	6.48E-05	0.003514	0.008593	0.137549
GR_C	19	0.882981	0.003062	4.85E-05	0.001153	0.000207	0.000154	0.00299	0.006787	0.102618
GR_D	20	0.859674	0.000472	0.000498	0.001733	0.000741	0.001306	0.001056	0.008396	0.126124
GR_X	21	0.845759	0.007651	0.000193	0.001407	0.000889	0.000205	0.001792	0.012198	0.129907
GR_Z	22	0.881456	0.003749	0.000423	0.001109	0.001202	0.003494	0.001191	0.009682	0.097693
PU_A	23	0.852391	0.010761	0.003005	0.005323	0.006283	0.000297	0.002064	0.007371	0.112504
PU_B1	24	0.751593	0.023829	0.006019	0.017063	0.016839	0.001189	0.003246	0.010922	0.169299
PU_B2	25	0.787325	0.001107	0.003724	0.002491	0.003399	0.000248	0.00107	0.007978	0.192658
PU_C	26	0.880289	0.004126	0.000165	0.00071	0.000641	0.000146	0.002308	0.008995	0.10262
PU_D	27	0.842887	0.000677	0.000172	0.000527	0.000432	0	0.002215	0.009184	0.143904
GR_JU	28	0	0.482307	0.00442	0.176727	0.336546	0	0	0	0
PU_JU	29	0	0.480405	0.004311	0.189355	0.325929	0	0	0	0
BL_JU	30	0	0.197558	0.010707	0.081403	0.710332	0	0	0	0
YE_A	31	0.866741	0.003811	0.000152	0.002147	0.000475	0.00014	0.002185	0.007706	0.116645
YE_B	32	0.874656	0.002608	0.000558	0.002229	0.002541	0.000208	0.001998	0.006538	0.108663
YE_D	33	0.848621	0.004756	0.000683	0.002368	0.004355	0.000365	0.000834	0.010084	0.127935
YE_JU	34	0	0.546603	0.001841	0.127177	0.324379	0	0	0	0
YE_Z	35	0.916672	0.001544	0.000343	0.001052	0.001517	0.000116	0.002794	0.00681	0.069152
YE_B2	36	0.874656	0.002608	0.000558	0.002229	0.002541	0.000208	0.001998	0.006538	0.108663

Table S6. Eigenvalues and variance explained by the principal components resulting from the REL analysis.

PC	1	2	3	4	5	6	7	8	9
Eigenvalue	4.744784	2.428596	0.972154	0.372068	0.276201	0.186960	0.015201	0.004036	1.49E-31
% of variance	52.72	26.98	10.80	4.13	3.07	2.08	0.17	0.04	0.00
sum % of variance	52.72	79.70	90.51	94.64	97.71	99.79	99.96	100.00	100.00

Table S7. Loadings of the original variables in the space of the first three varivectors (dimensions) resulting from the **REL** analysis. One can calculate the coordinates of the samples in space of the first three varivectors by multiplying matrix presented in Table S4 by the autoscaled version of the matrix presented in Table S6.

	**Dim1**	**Dim2**	**Dim3**
G2	-0.885	-0.433	0.064
G6	0.897	0.281	-0.096
G9	0.137	0.960	0.171
G7	0.627	0.760	0.020
G5	0.912	-0.023	-0.093
G4	-0.057	0.300	0.922
G8	-0.764	0.272	-0.402
G1	-0.062	0.969	0.167
G3	-0.871	-0.073	-0.029

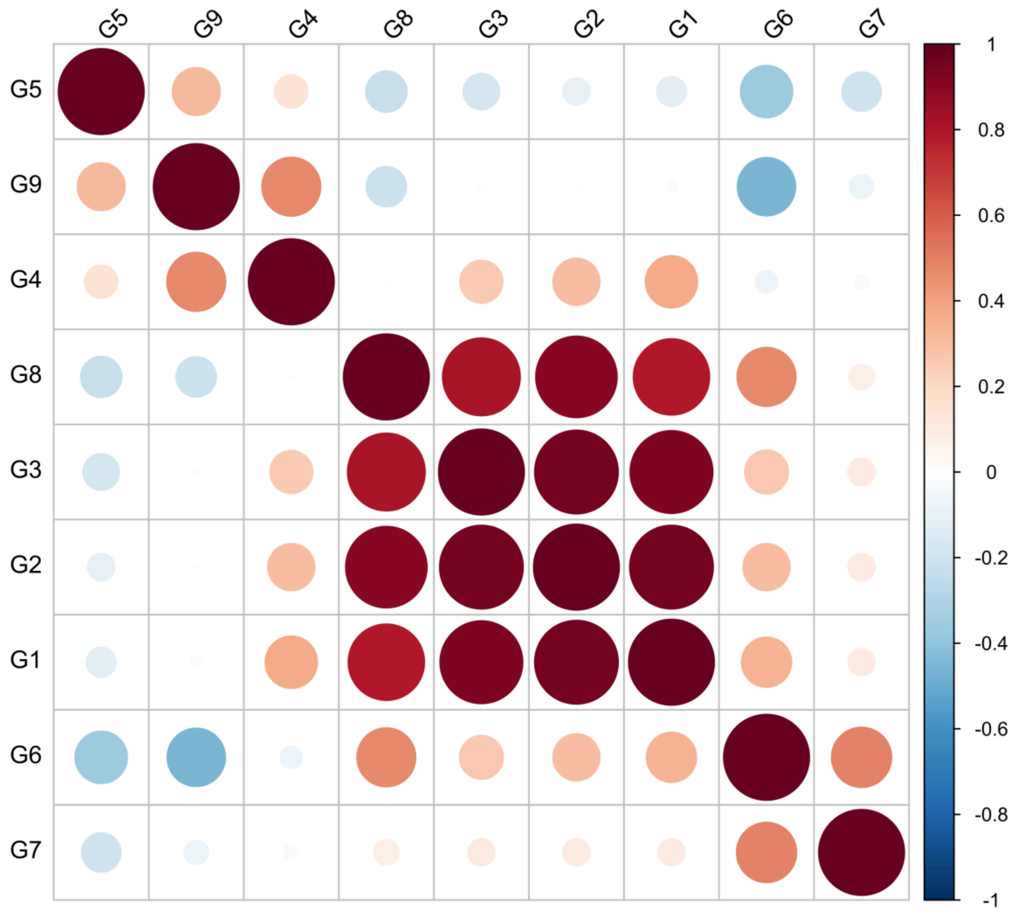

Figure S4. Graphical representation of the correlation coefficients (R) for the ABS dataset.

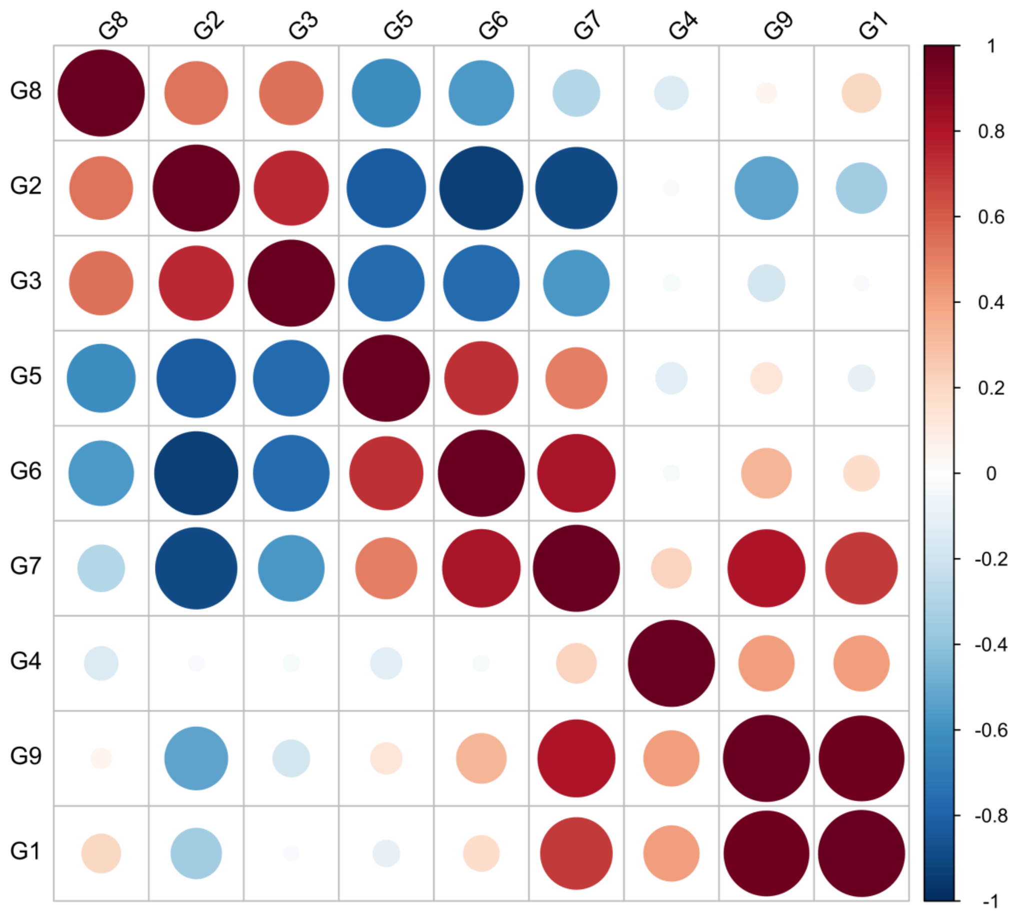

Figure S5. Graphical representation of the correlation coefficients (R) for the REL dataset.
